# Supplementary material for: Regioselective and Stereoselective Synthesis of Parthenolide Analogs by Acyl Nitroso-Ene Reaction and Their Biological Evaluation against Mycobacterium tuberculosis
Source: Int J Mol Sci. 2023 Dec 12;24(24):17395. doi: 10.3390/ijms242417395 (PMC10744032; doi:10.3390/ijms242417395)
Supplement: Supplementary file 1 [file ijms-24-17395-s001.zip › ijms-2727802-supplementary.pdf]

Regioselective and Stereoselective Synthesis of Parthenolide Analogs by Acyl Nitroso-Ene Reaction and Their Biological Evaluation against *Mycobacterium tuberculosis*

Bruna Gioia [1], Francesca Ruggieri [1], Alexandre Biela [1], Valérie Landry [1], Pascal Roussel [2], Catherine Piveteau [1], Florence Leroux [1], Ruben C. Hartkoorn [3] and Nicolas Willand [1,*]

[1] Univ. Lille, Inserm, Institut Pasteur de Lille, U1177—Drugs and Molecules for living Systems, F-59000 Lille, France
[2] Univ. Lille, CNRS, ENSCL, Centrale Lille, Univ. Artois, UMR 8181—UCCS—Unité de Catalyse et de Chimie du Solide, F-59000 Lille, France
[3] Univ. Lille, CNRS, Inserm, CHU Lille, Institut Pasteur de Lille, U1019—UMR 8204—CIIL—Center for Infection and Immunity of Lille, F-59000 Lille, France
* Correspondence: nicolas.willand@univ-lille.fr

Supplementary material

*Table S1. Summary of methodology for nitroso-ene reaction between parthenolide and 1-hydroxy-3-phenyl-urea (**2a**). Reaction was run on 50 mg of parthenolide besides condition S.*

	Parthenolide (eq.)	1-hydroxy-3-phenyl-urea (**eq.**)	Pyridine (eq.)	H_2O_2 30% in H_2O (eq.)	CuCl (eq.)	CuI (eq.)	Yield	Time	Conditions
A	1	1.1	0.25[a]	-	0.05	-	61%	48	Air
B[1]	1	1.1	0.125	-	0.1	-	N.D.	24	Air
C[1]	1	1.1	0.25	-	0.1	-	N.D.	24	Air
D	1	1.1	0.25	-	0.05	-	55%	48	Air
E	1	1.5	0.25	-	0.05	-	62%	24	Air
F	1	1.1	-	0.8		0.05	32%	24	Air
G[1]	1	1.1	0.125 + 0.125 (after 24h)	-	0.05 + 0.05 (after 24h)	-	N.D.	48	Air
H[1]	1	1.1	0.1	-	0.1	-	N.D.	48	Air
I[1]	1	1.1	-	0.8	0.05	-	N.D.	48	Air
L[2]	1	1.1	0.125	-	0.05	-	N.D.	-	Microwave irradiation
M	1	1.1	0.125	-	0.05	-	69%	24	O_2 atmosphere
N	1	1.5	0.25	-	0.05	-	50%	24	O_2 atmosphere
O	1	1.5	0.125	-	0.05	-	80%	24	O_2 atmosphere
R[1]	1	2	0.125	-	0.05	-	N.D.	24	O_2 atmosphere
S[3]	1	1.1	0.0375	-	0.01	-	43%	48	Air

[1] Complex mixture with formation of side compounds, compound **3a** was not isolated. [2] Reaction was run under microwave irradiation (µW) for 45 min at 50°C, then for 1h30at 65 °C but conversion was not total and yield was not calculated, compound **3a** was not isolated. [3] Reaction was run on 500 mg.

Materials

NMR spectra were recorded on a Bruker® Avance-300 spectrometer (Wissembourg, France). The results were calibrated to signals from the solvent as an internal reference (e.g., 7.26 (residual $CDCl_3$) and 77.16 ($CDCl_3$) ppm and 2.50 (residual DMSO d^6) and 39.52 (DMSO d^6) ppm for ^1H and ^{13}C NMR spectra, respectively.) Chemical shifts (δ) are in parts per million (ppm) downfield from tetramethylsilane (TMS). The assignments were made using one-dimensional (1D) ^1H and ^{13}C spectra and two-dimensional (2D) HSQC-DEPT, COSY, and HMBC spectra. NMR coupling constants (J) are reported in hertz (Hz), and splitting patterns are indicated as follows: s (singlet); brs (broad singlet); d (doublet); dd (doublet of doublet); ddd (double of doublet of doublet); dt (doublet of triplet); t (triplet); td (triplet of doublet); q (quartet); m (multiplet).

NMR spectral data

1-hydroxy-3-phenyl-urea (**2a**)
C7H8N2O2

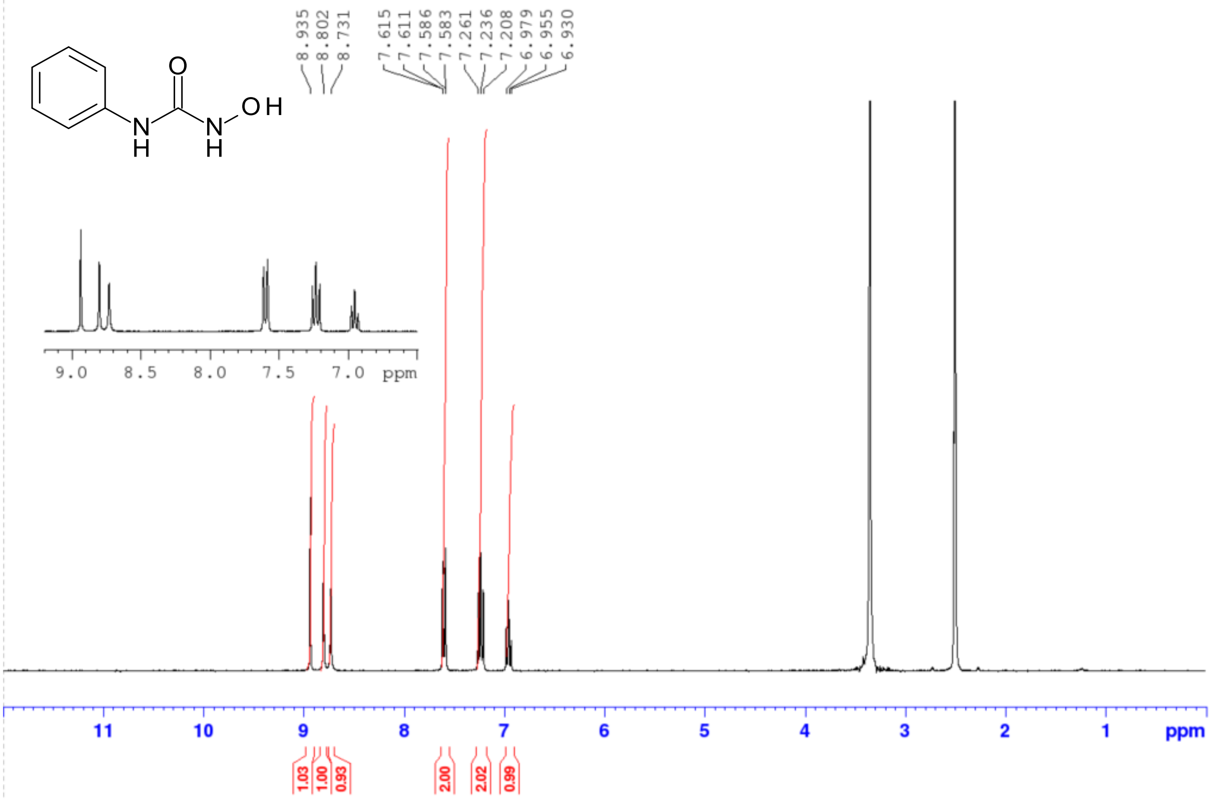

1-hydroxy-3-(p-tolyl)urea (**2b**)
C8H10N2O2

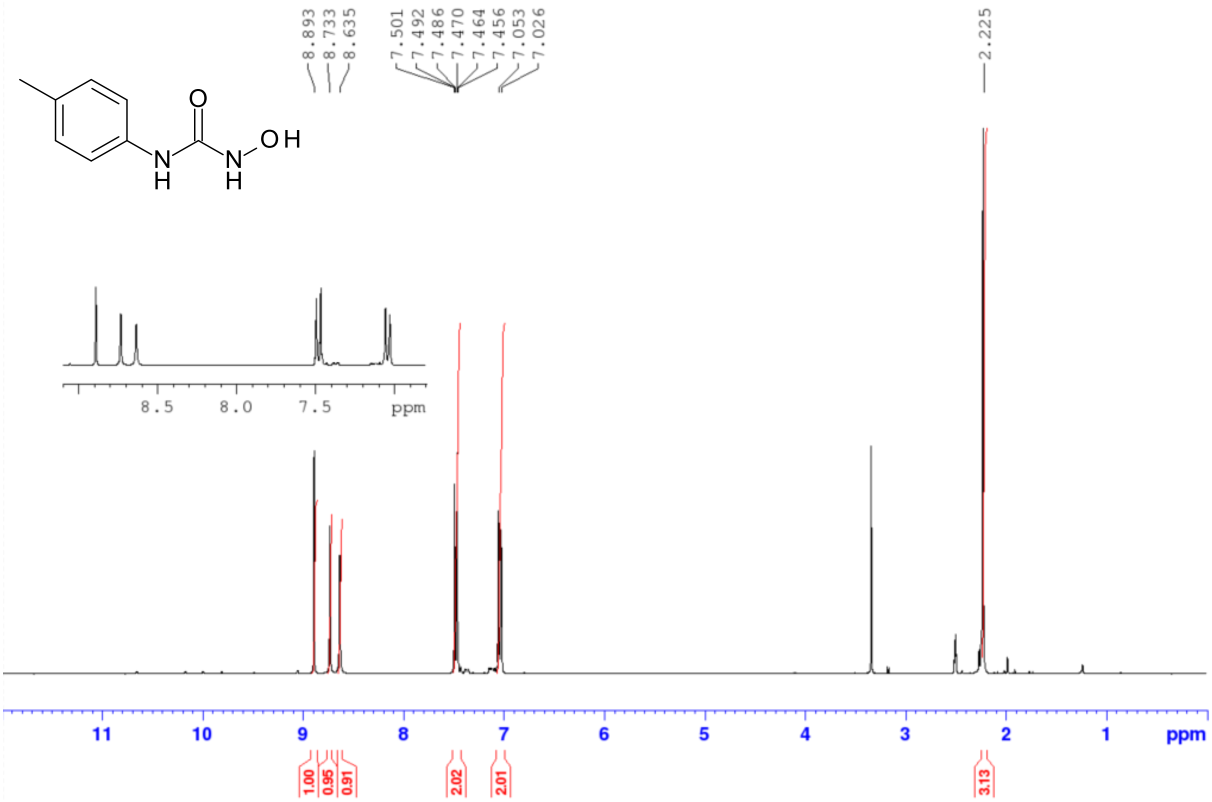

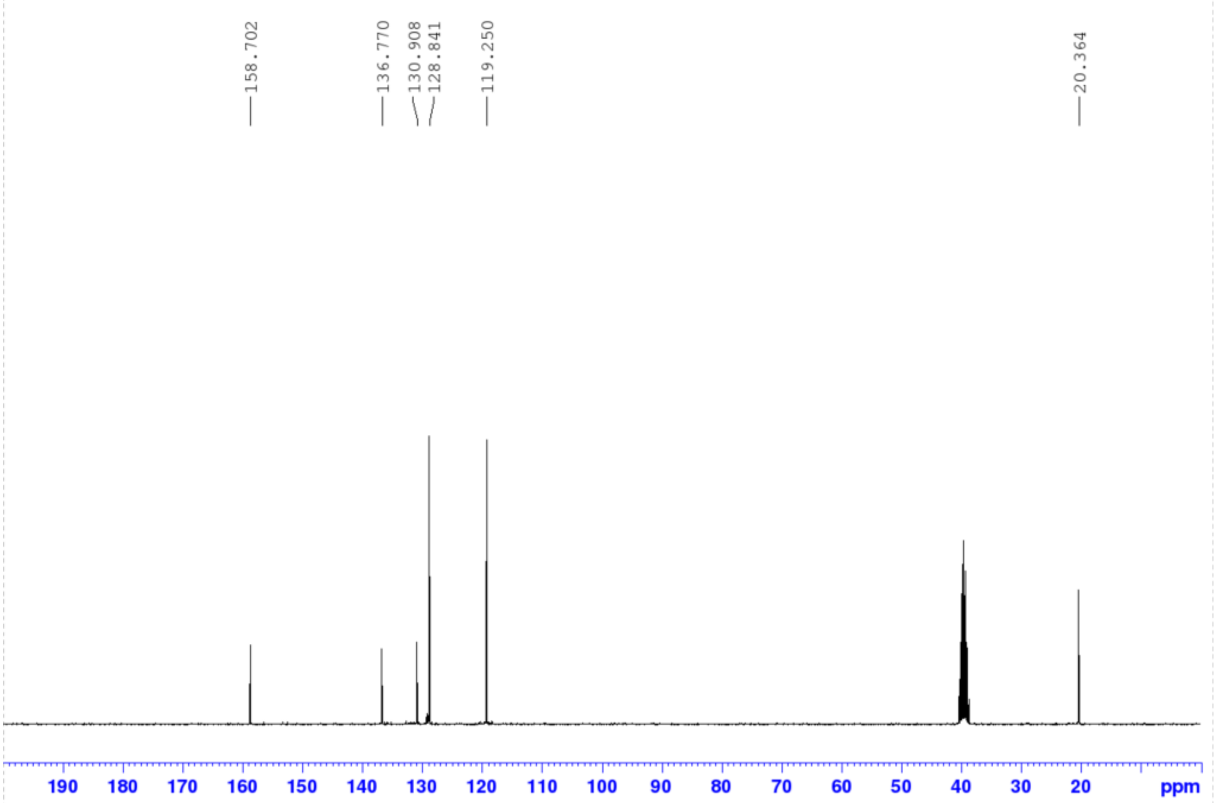

1-benzyl-3-hydroxy-urea (**2c**)
C8H10N2O2

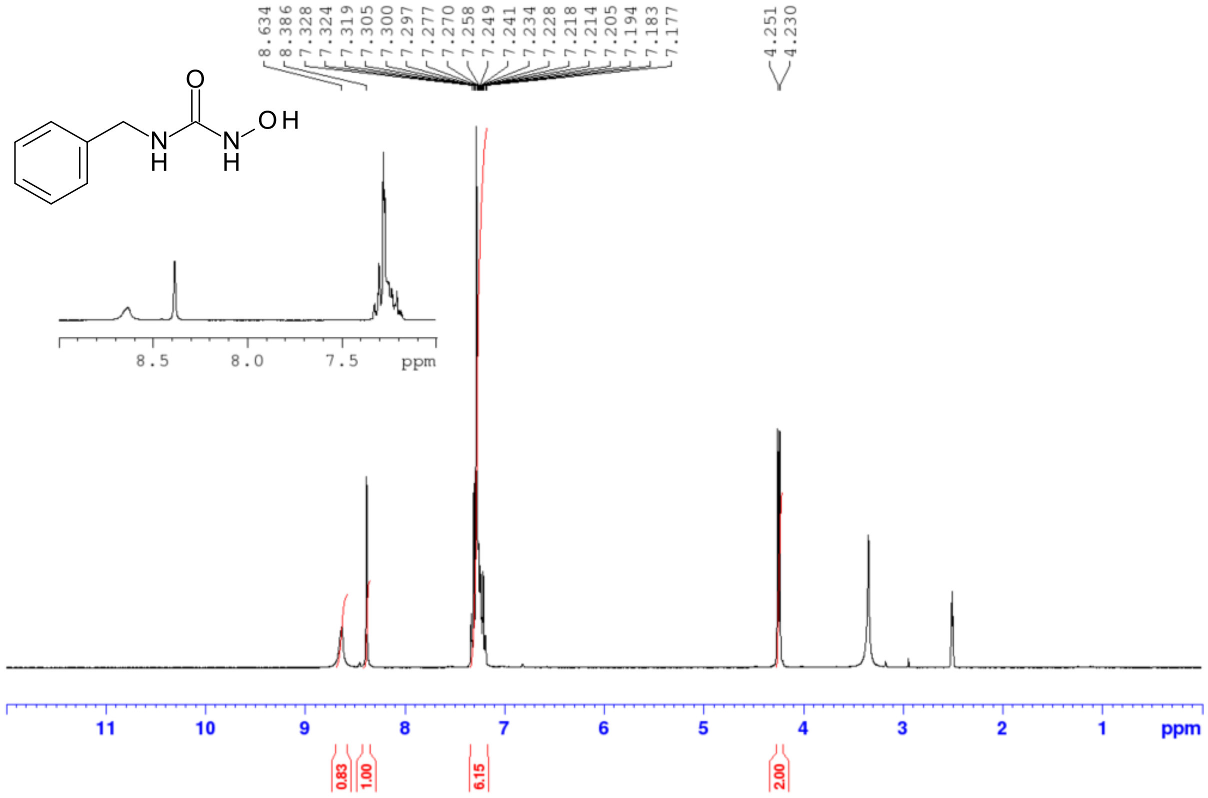

1-hydroxy-3-(4-methoxyphenyl)urea (**2d**)

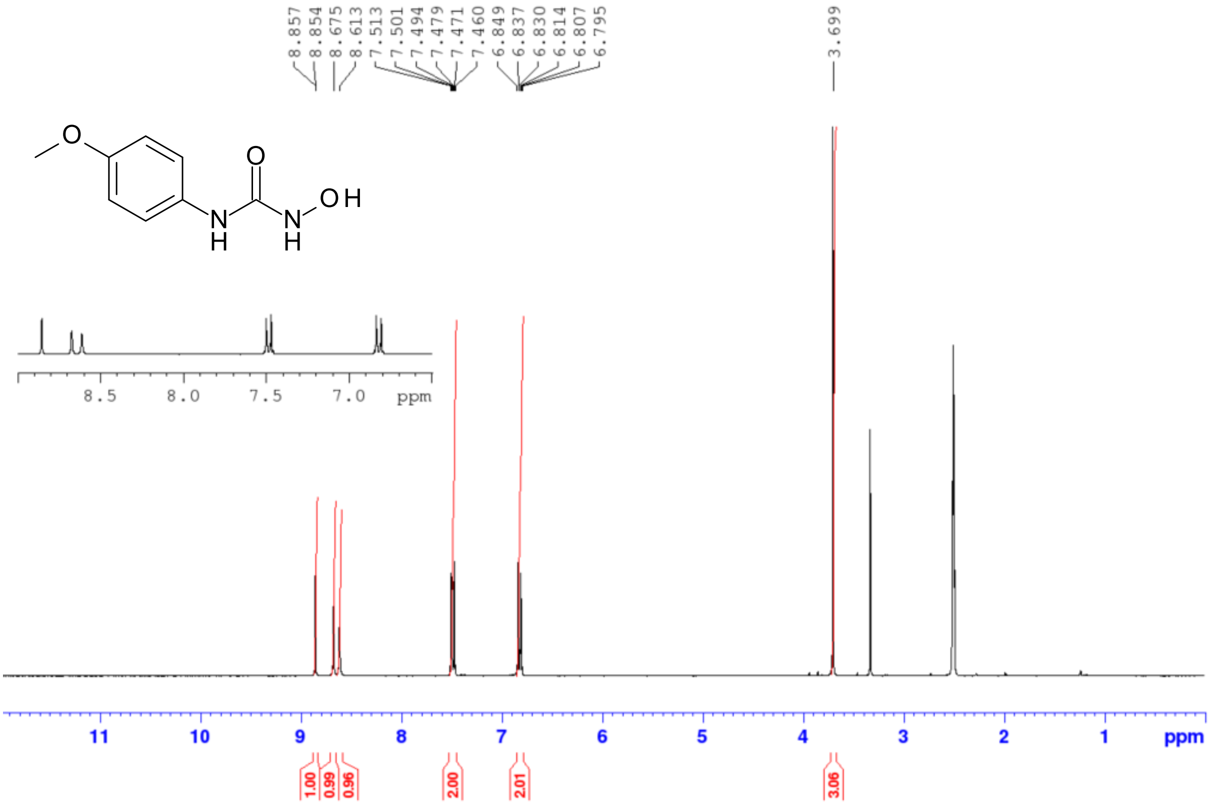

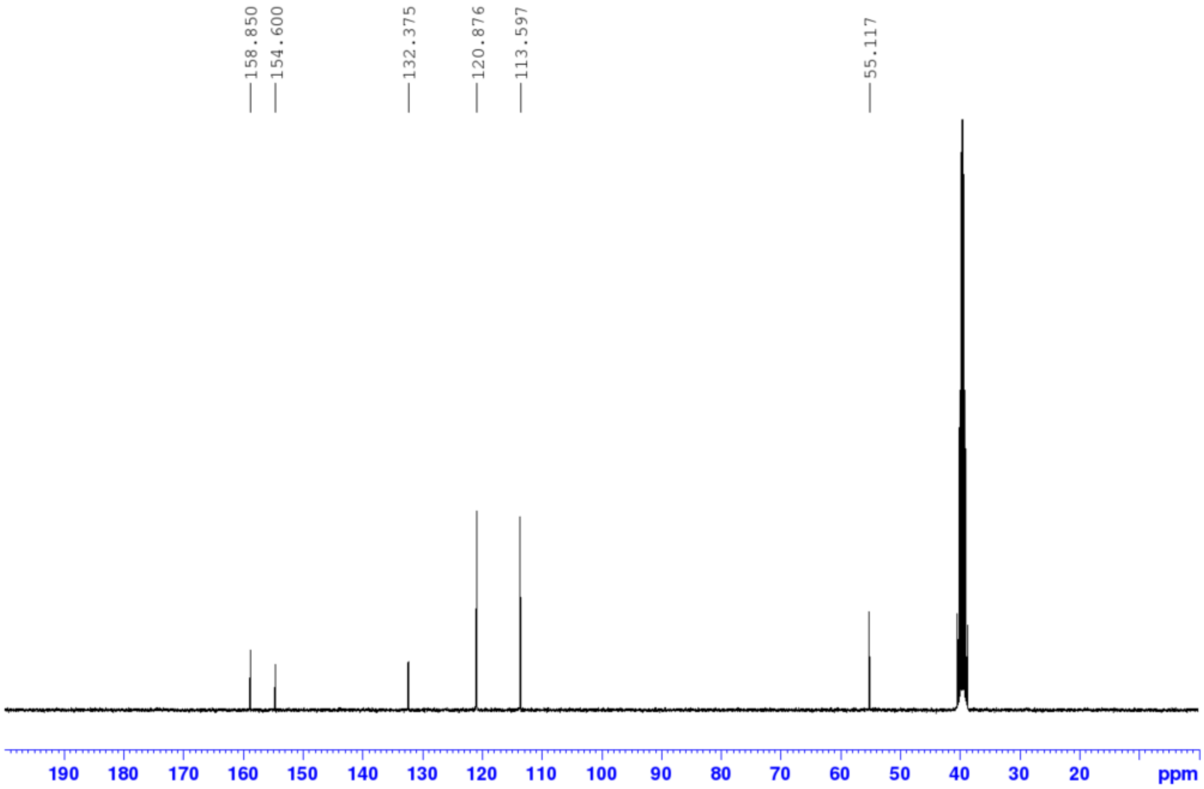

1-(4-fluorophenyl)-3-hydroxy-urea (**2e**)

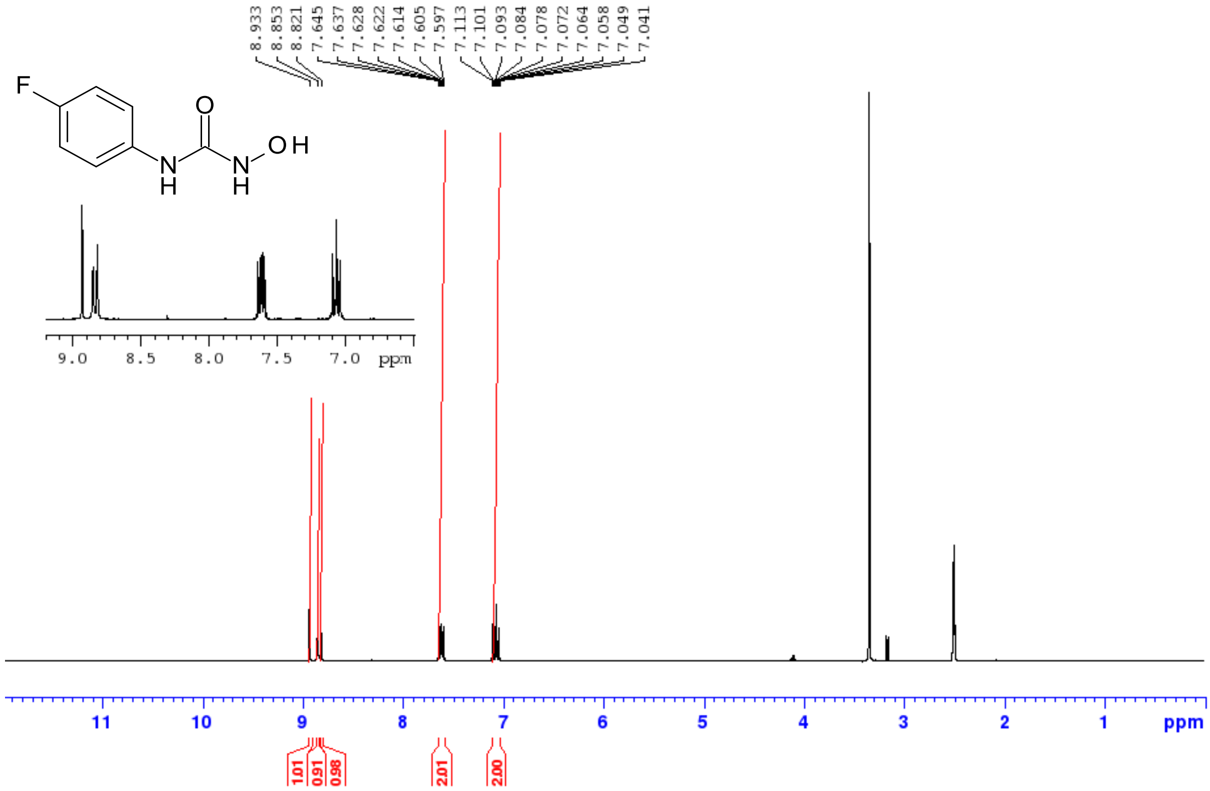

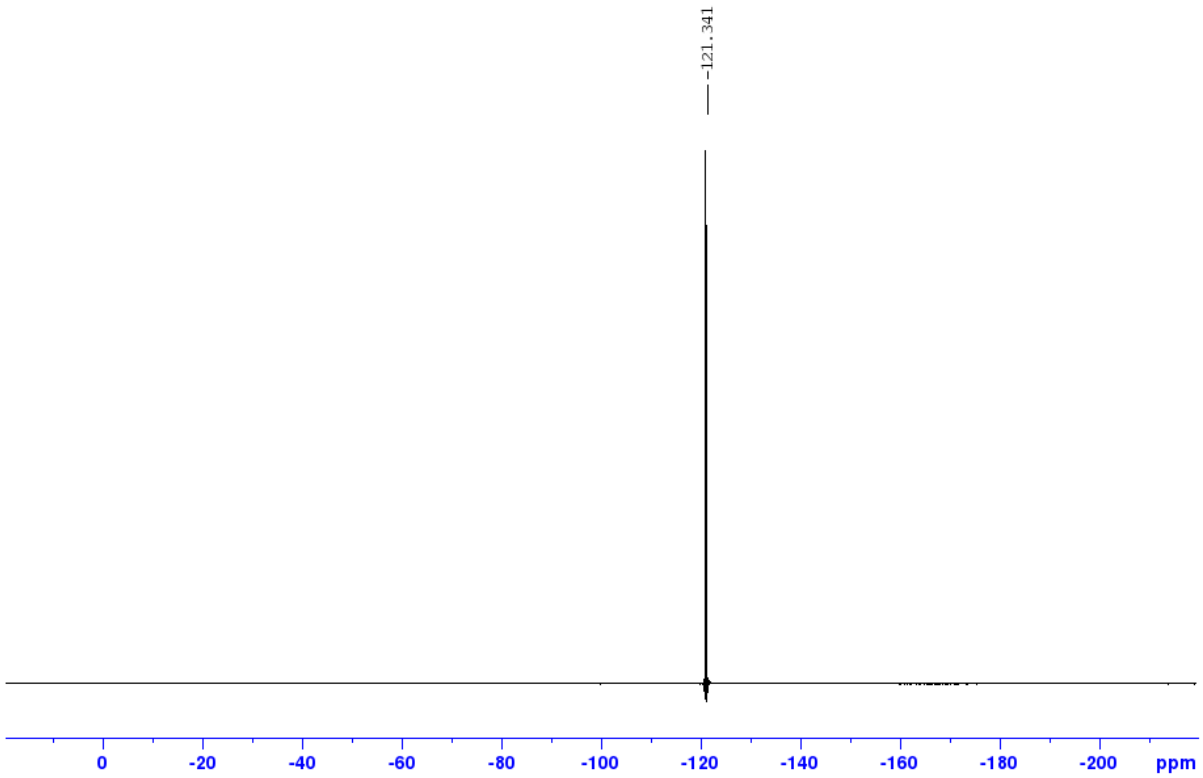

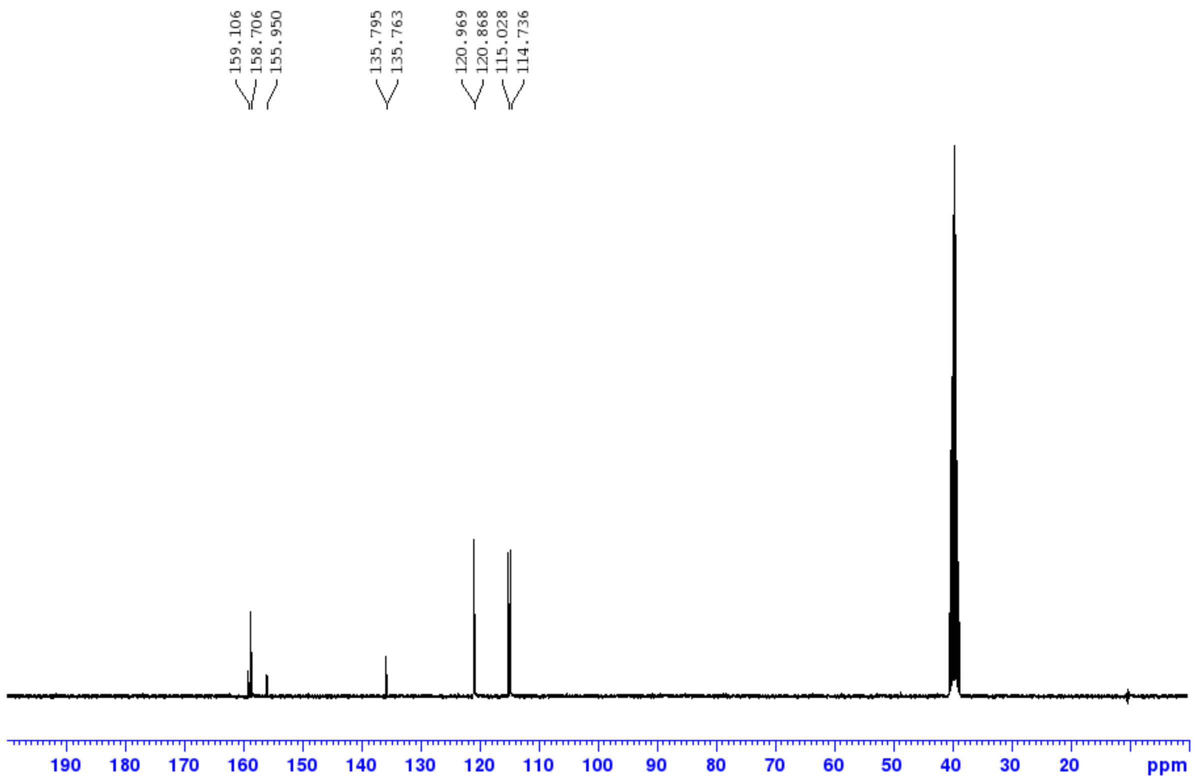
159.106
158.706
155.950
135.795
135.763
120.969
120.868
115.028
114.736
190 180 170 160 150 140 130 120 110 100 90 80 70 60 50 40 30 20 ppm

1-(1,3-benzodioxol-5-yl)-3-hydroxy-urea (**2g**)

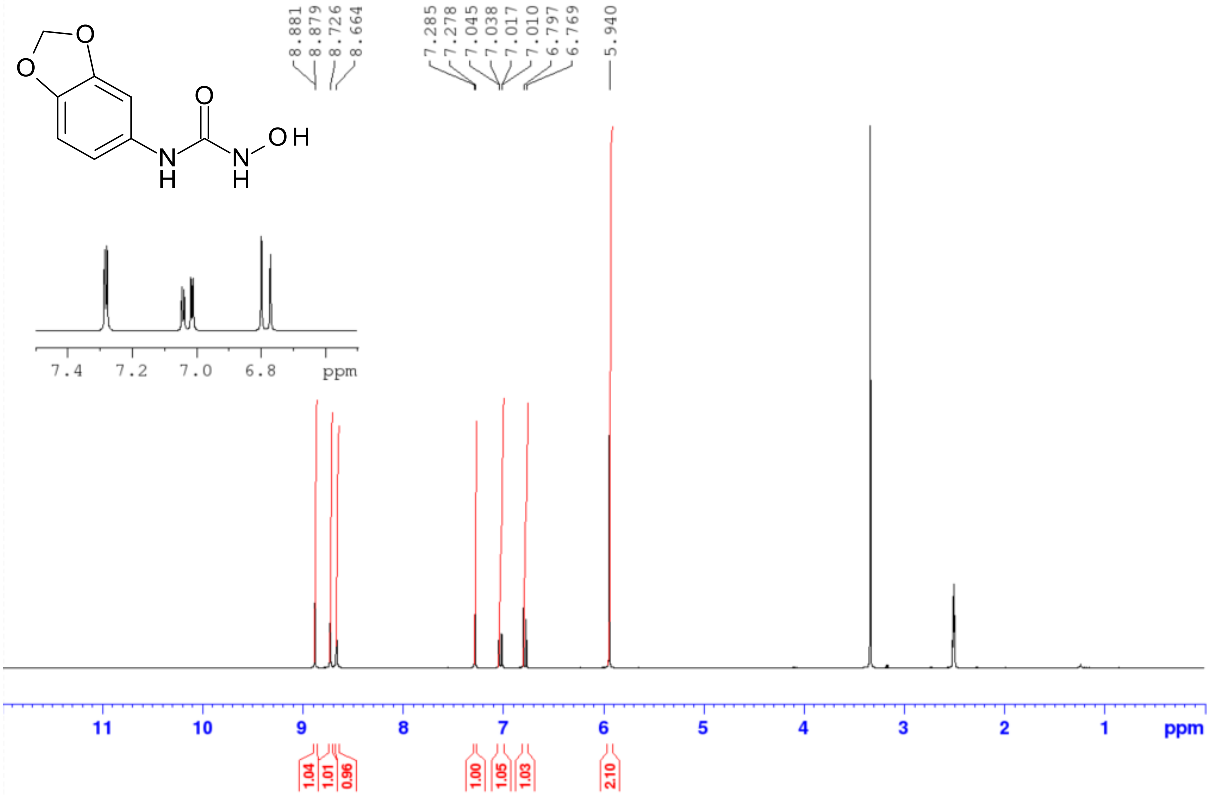

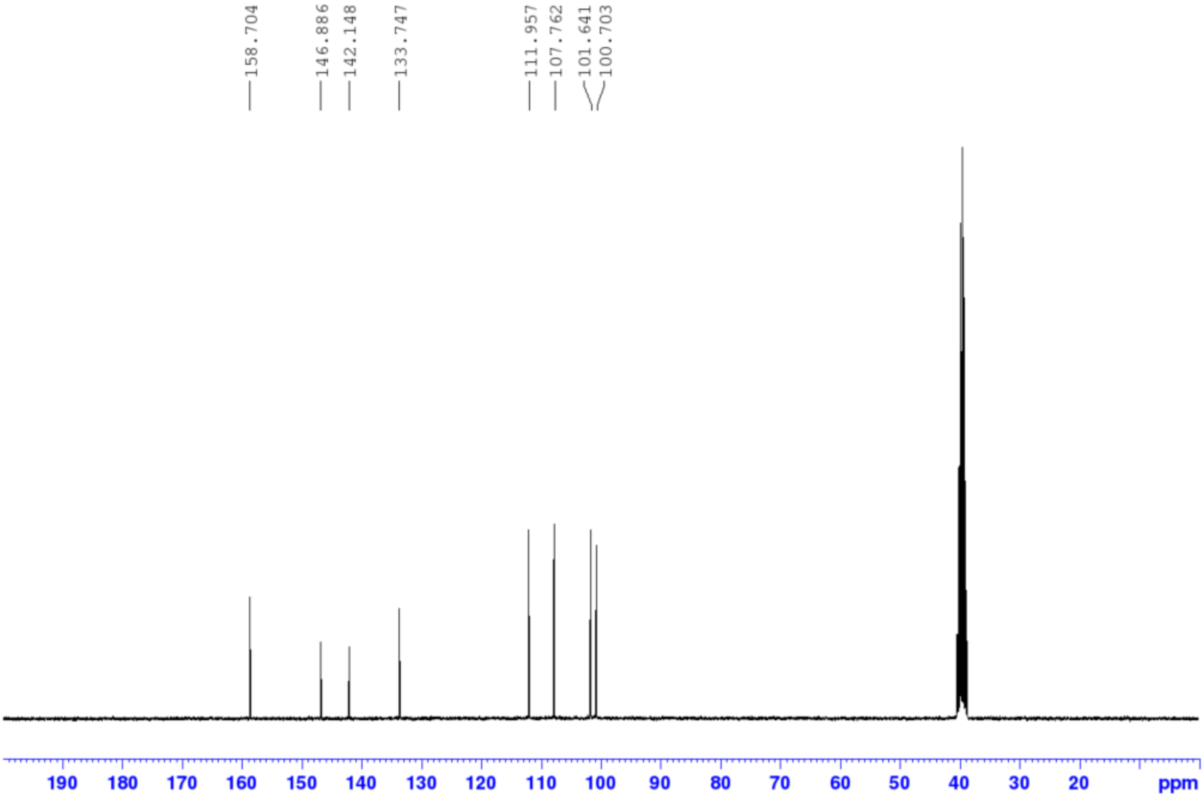

1-(3-ethoxypropyl)-3-hydroxy-urea (**2h**)

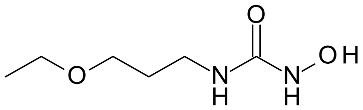

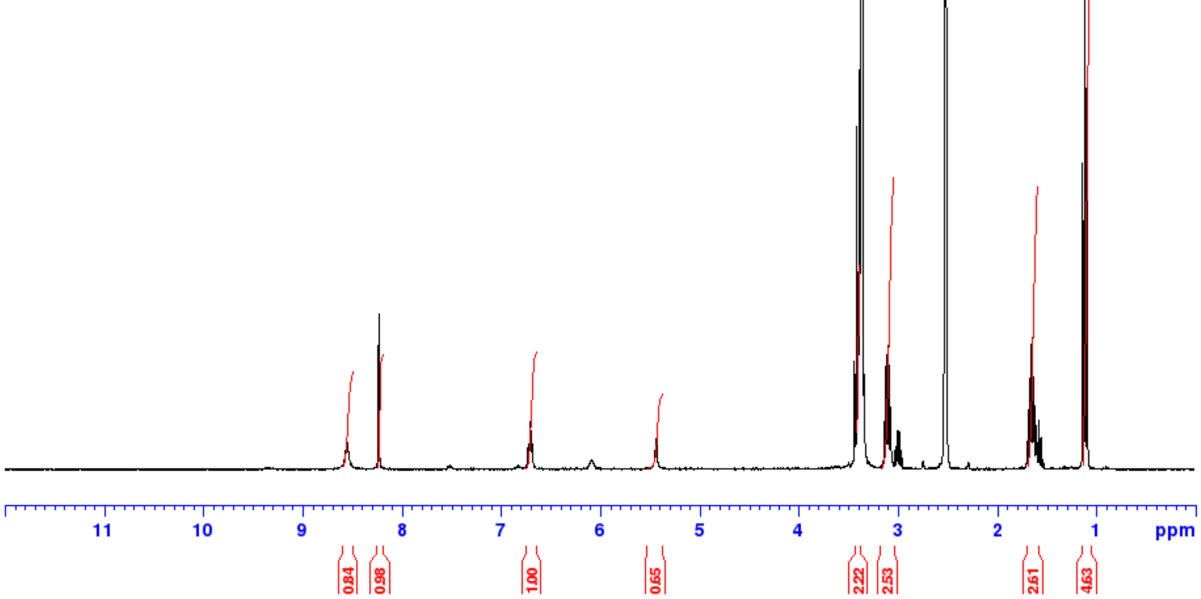

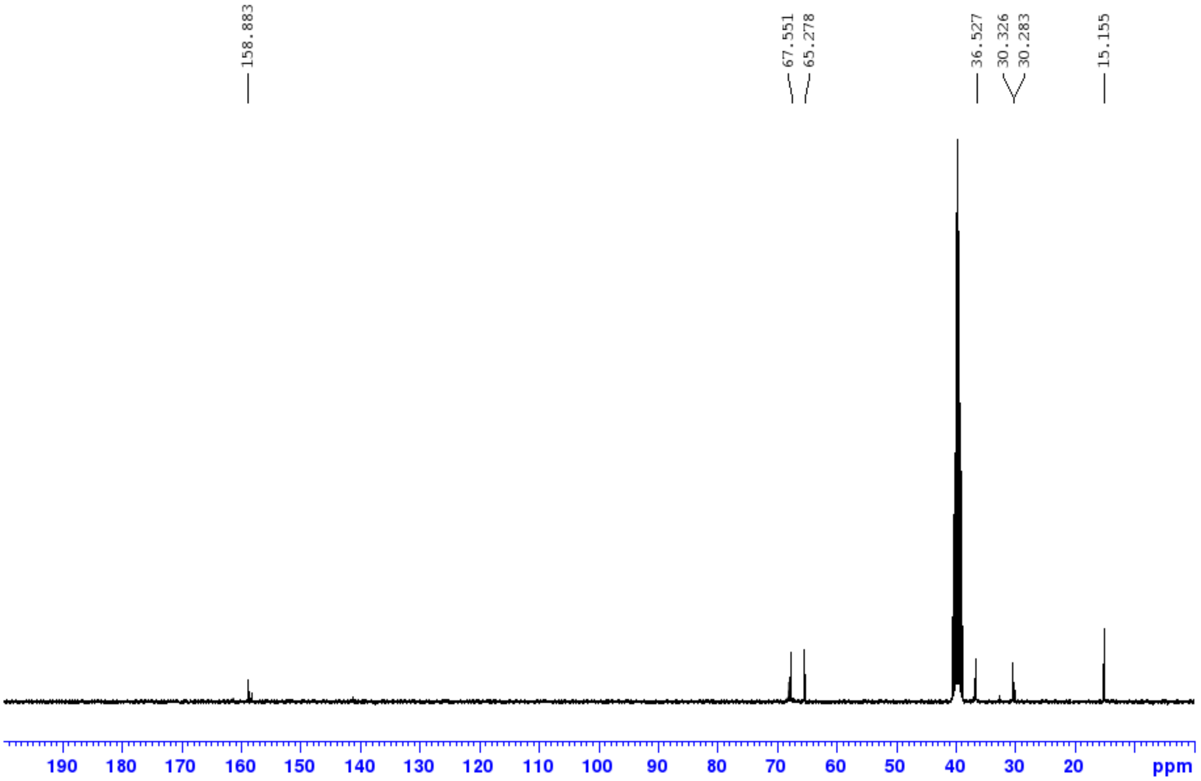

allyl N-hydroxycarbamate (**3b**)[1]

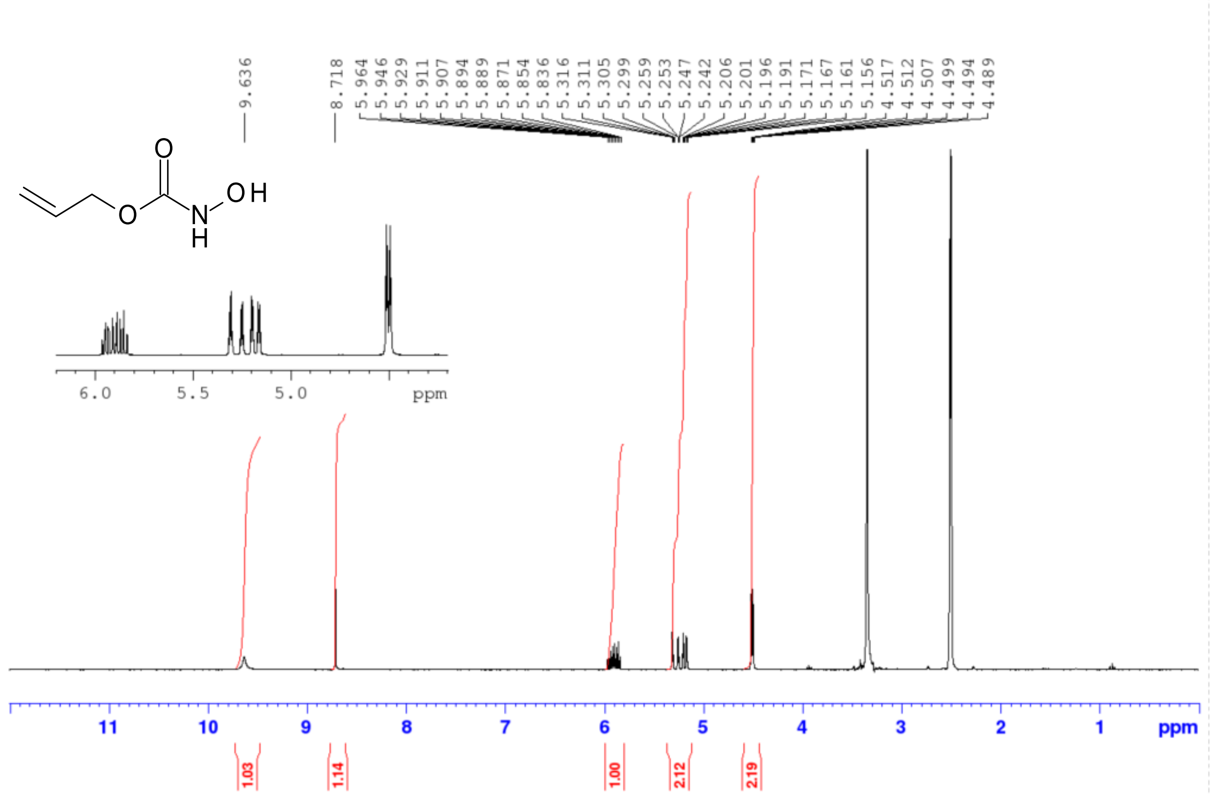

2,2,2-trichloroethyl N-hydroxycarbamate (**3d**)

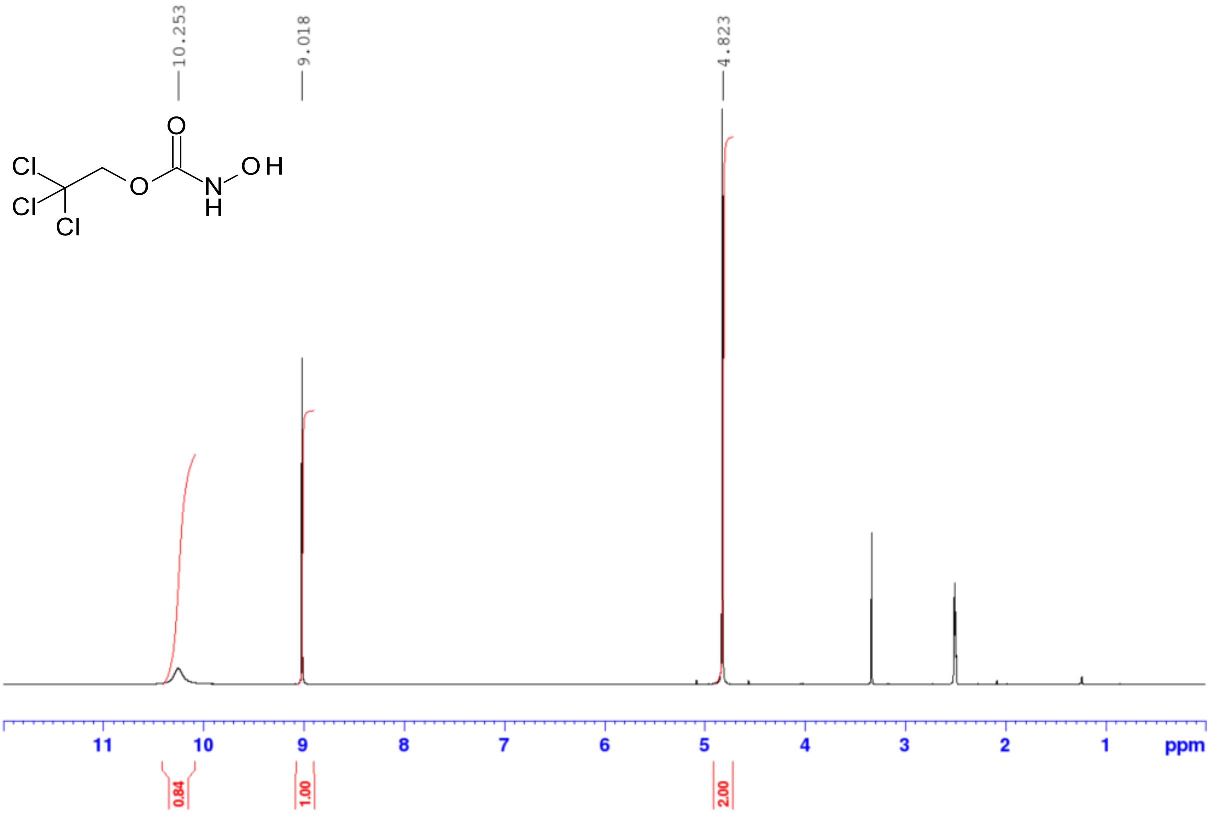

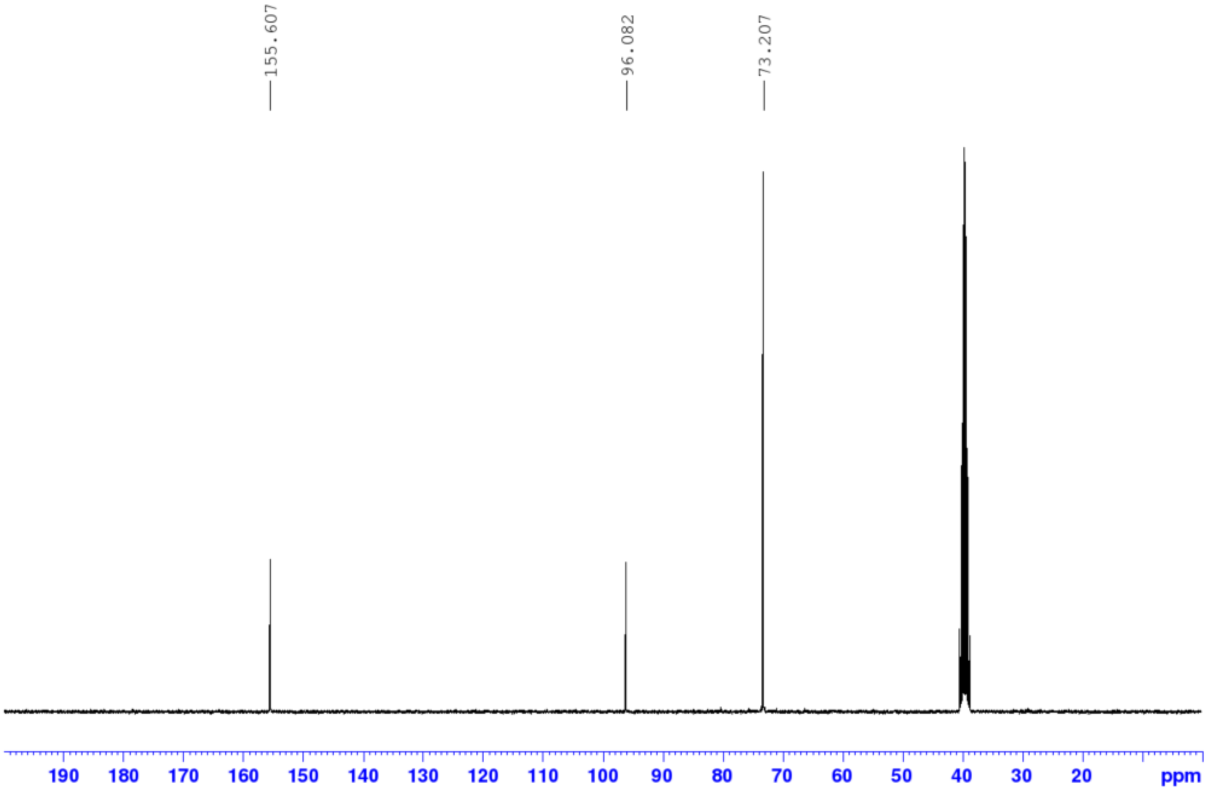

(4-fluorophenyl) N-hydroxycarbamate (**3e**)[2]

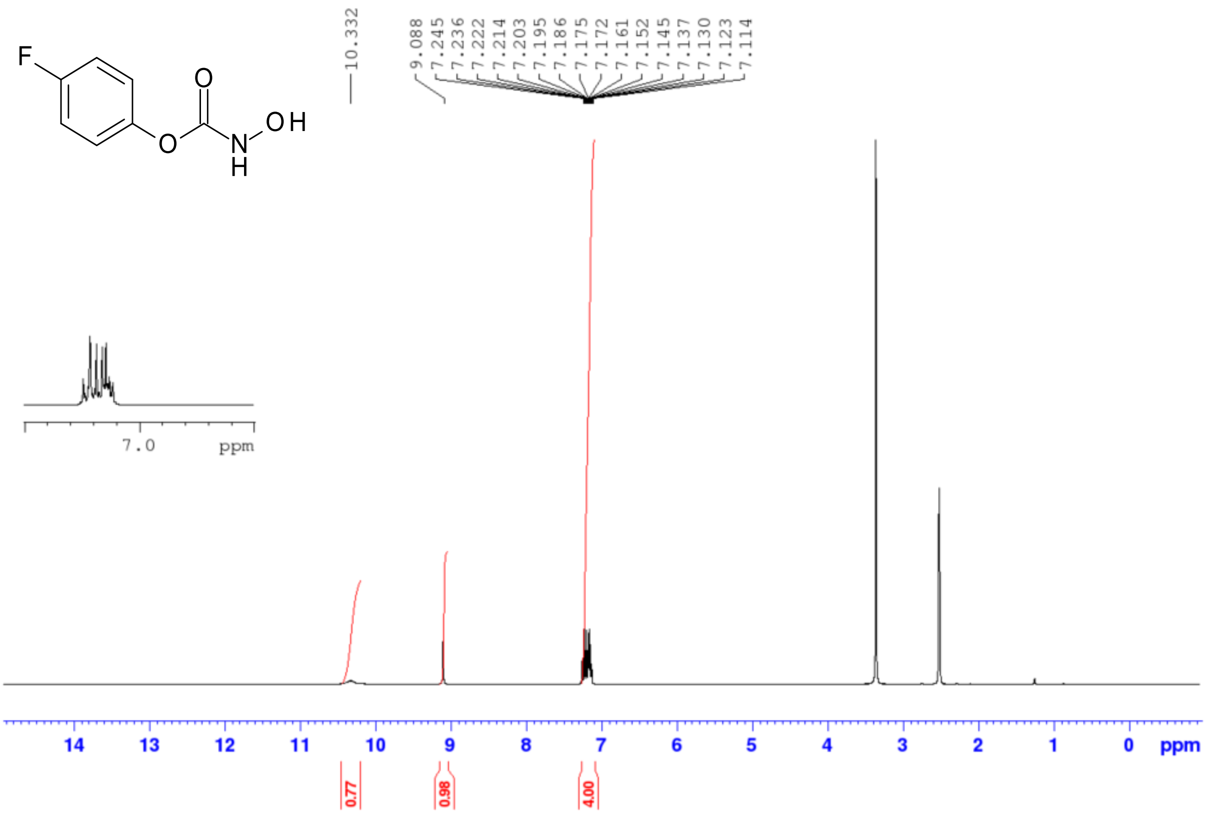

phenyl N-hydroxycarbamate (**3f**) [3]

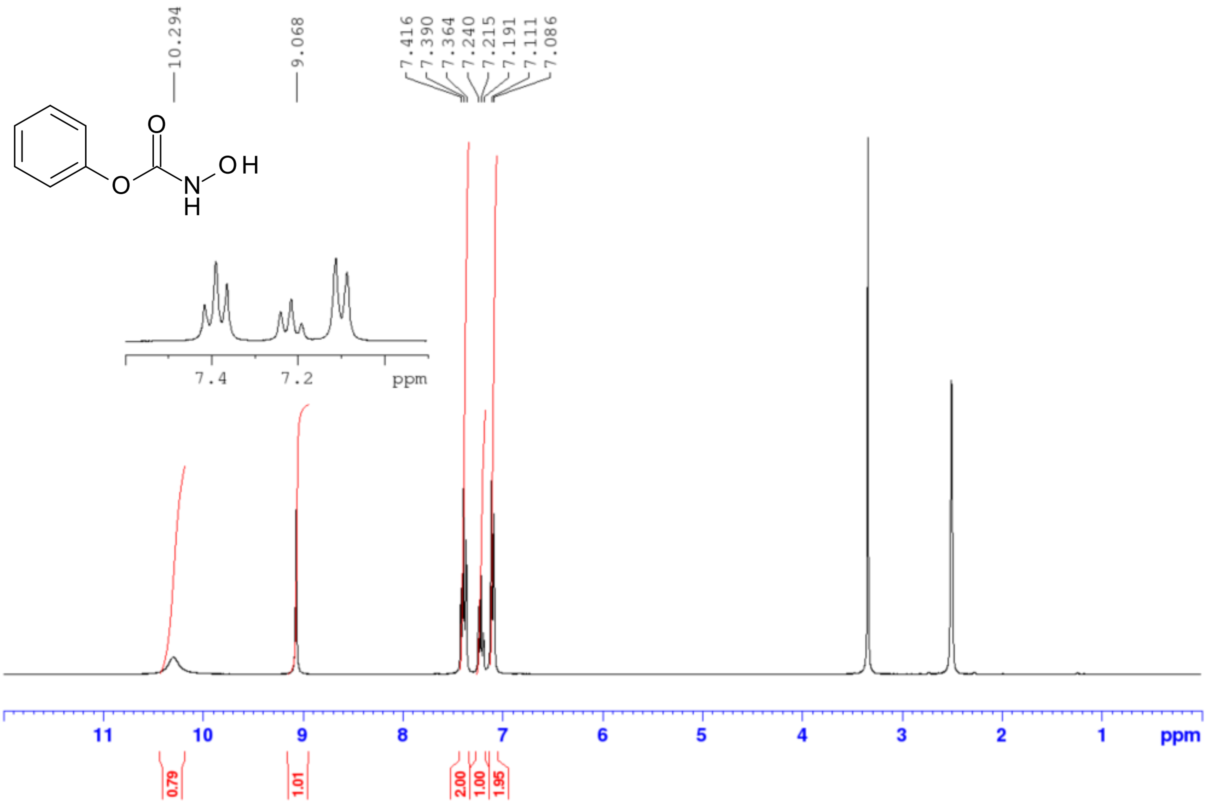

isobutyl N-hydroxycarbamate (**3g**)

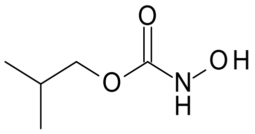

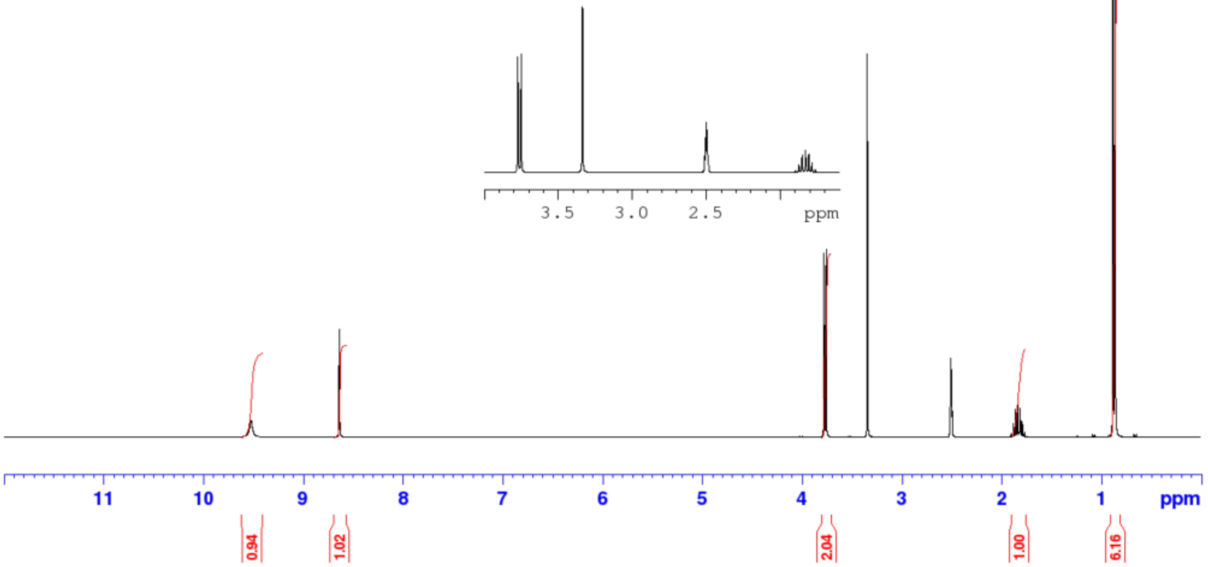

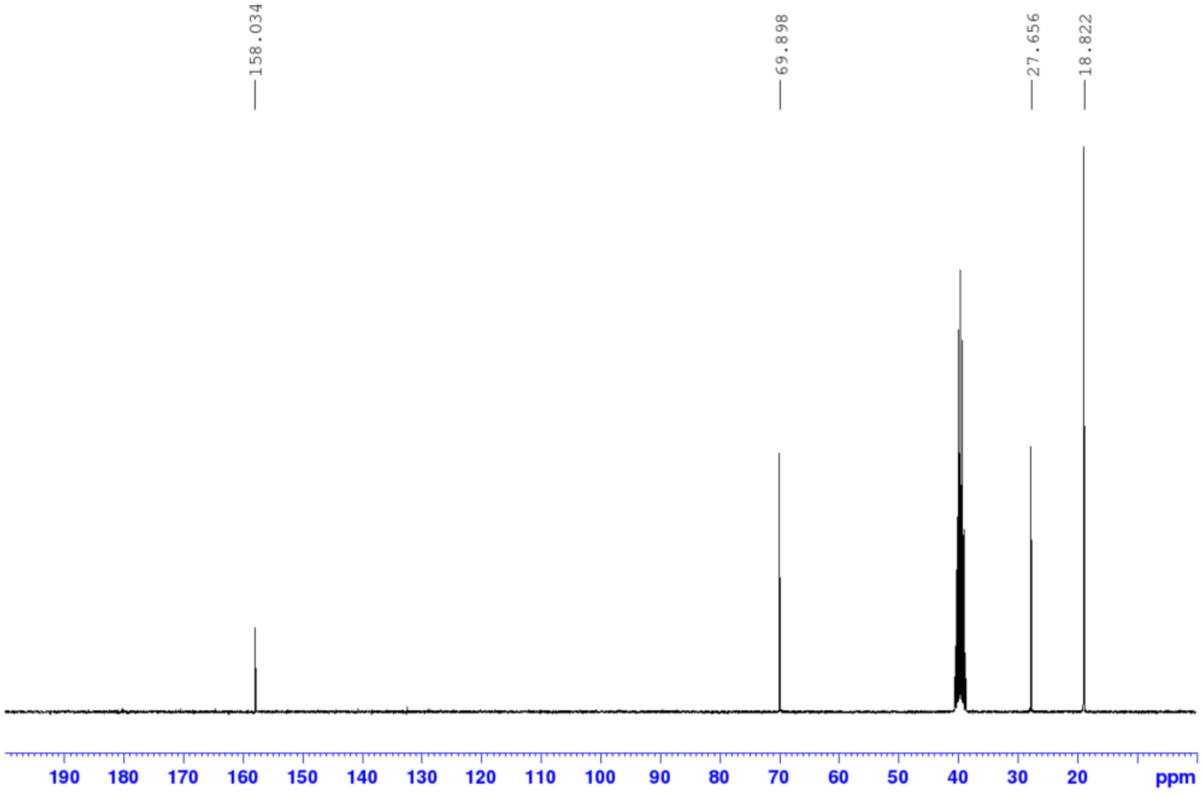

ethyl N-hydroxycarbamate (**3h**)[4]

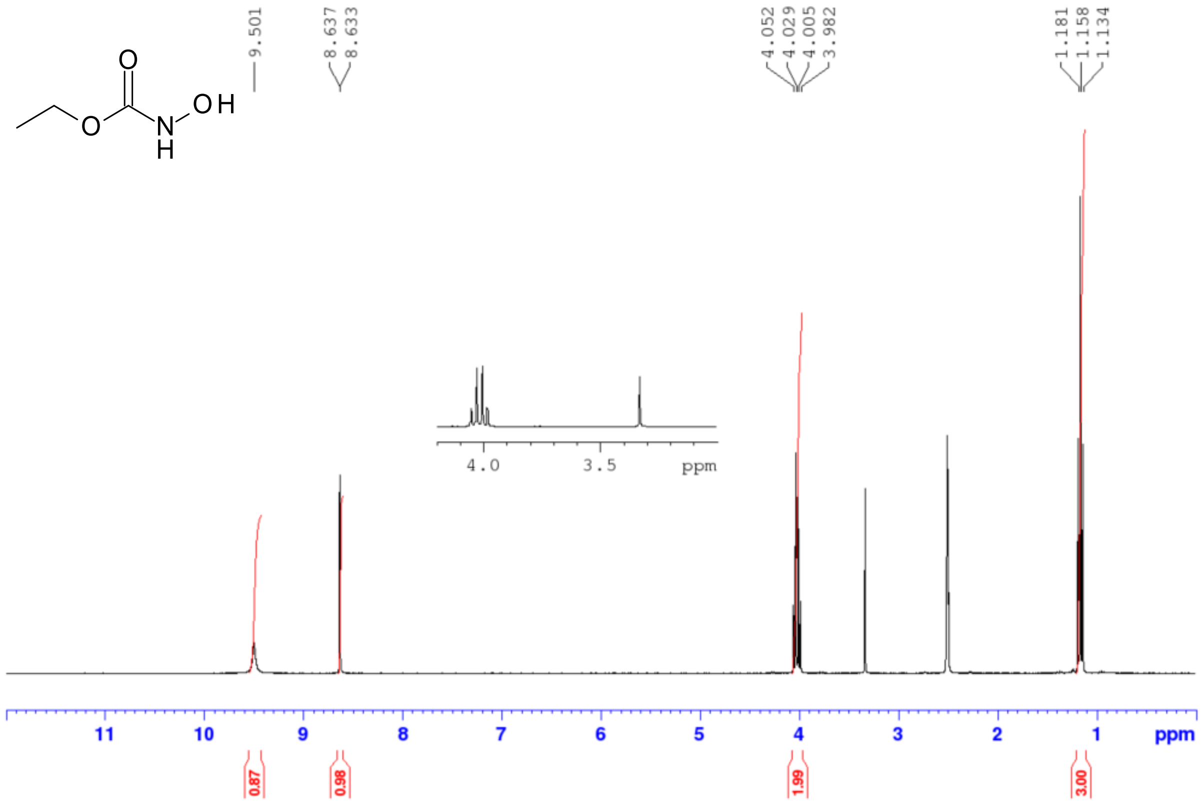

1,3-benzodioxol-5-yl N-hydroxycarbamate (**3i**)

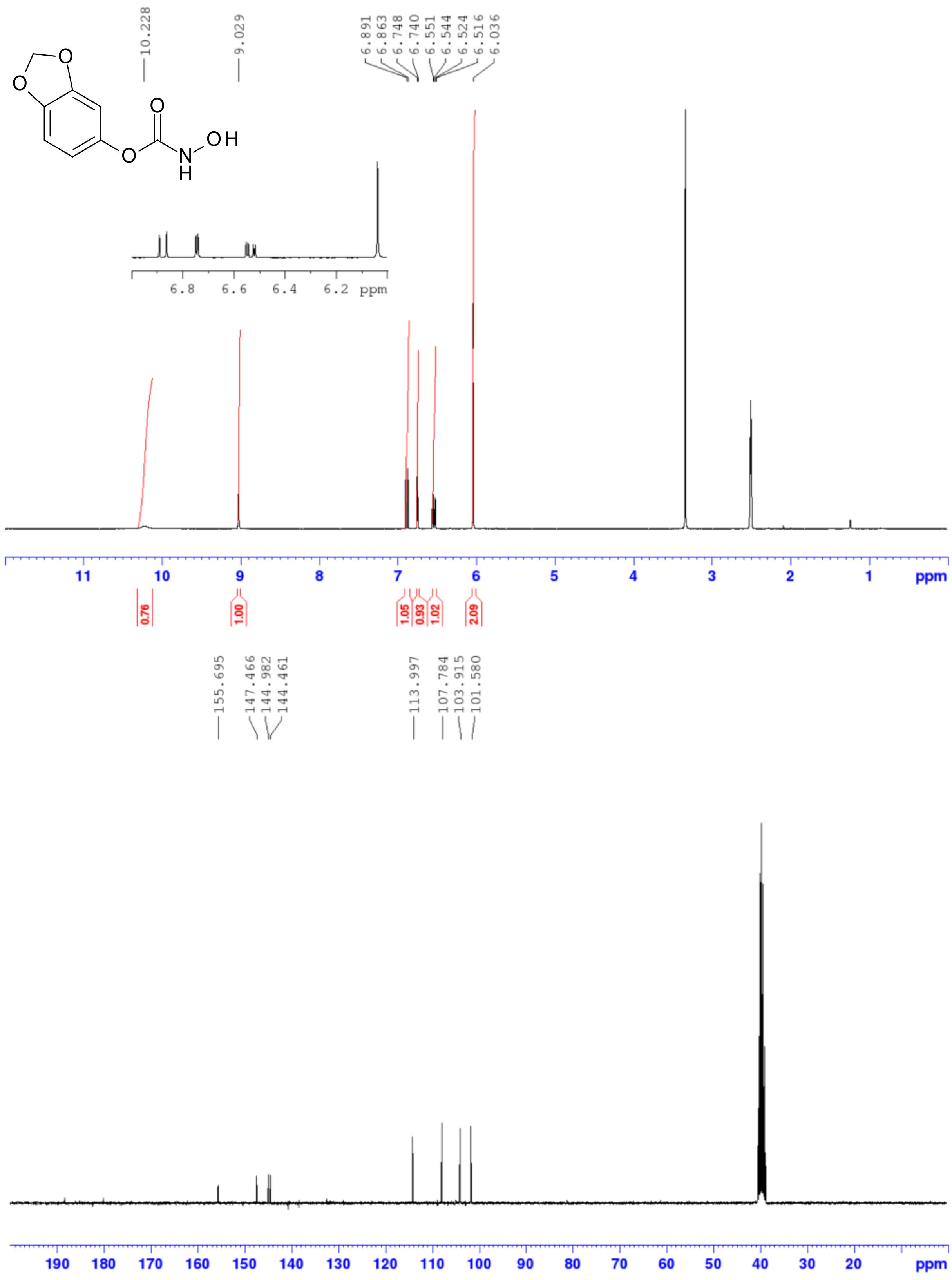

2-methoxyethyl N-hydroxycarbamate (**3j**)

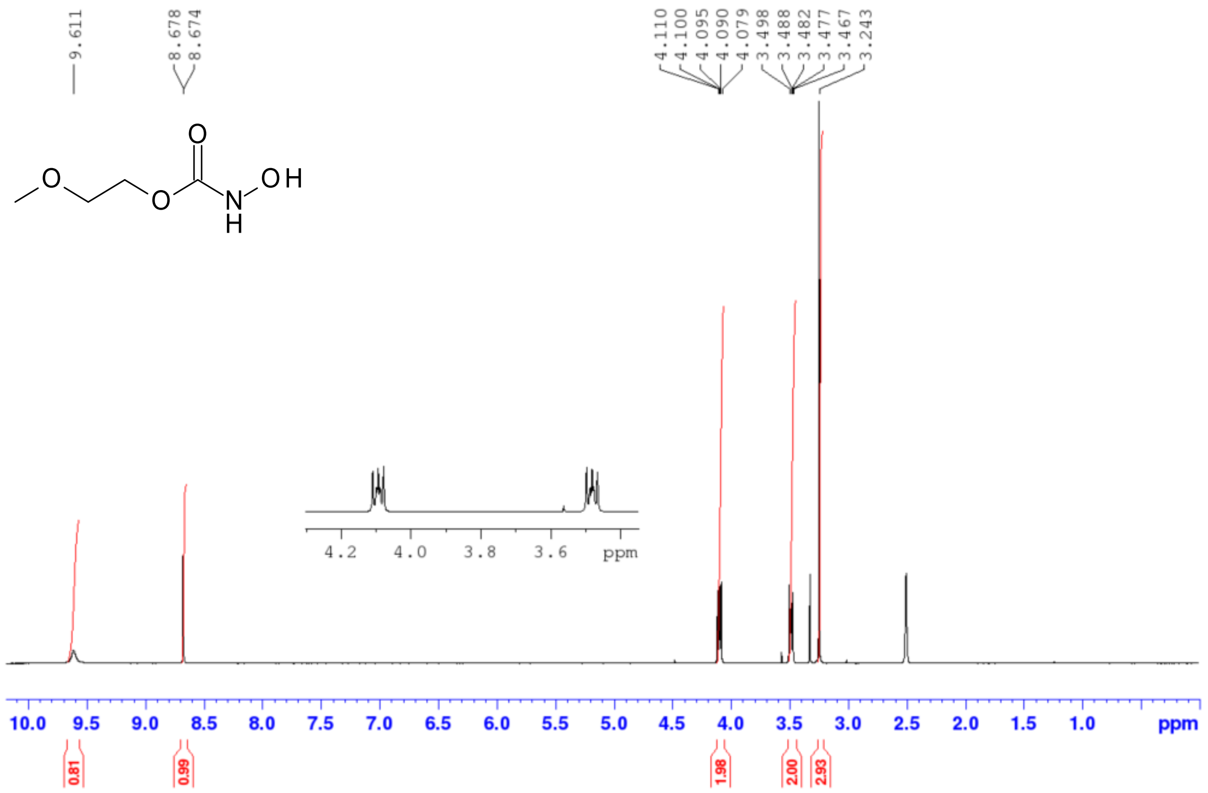

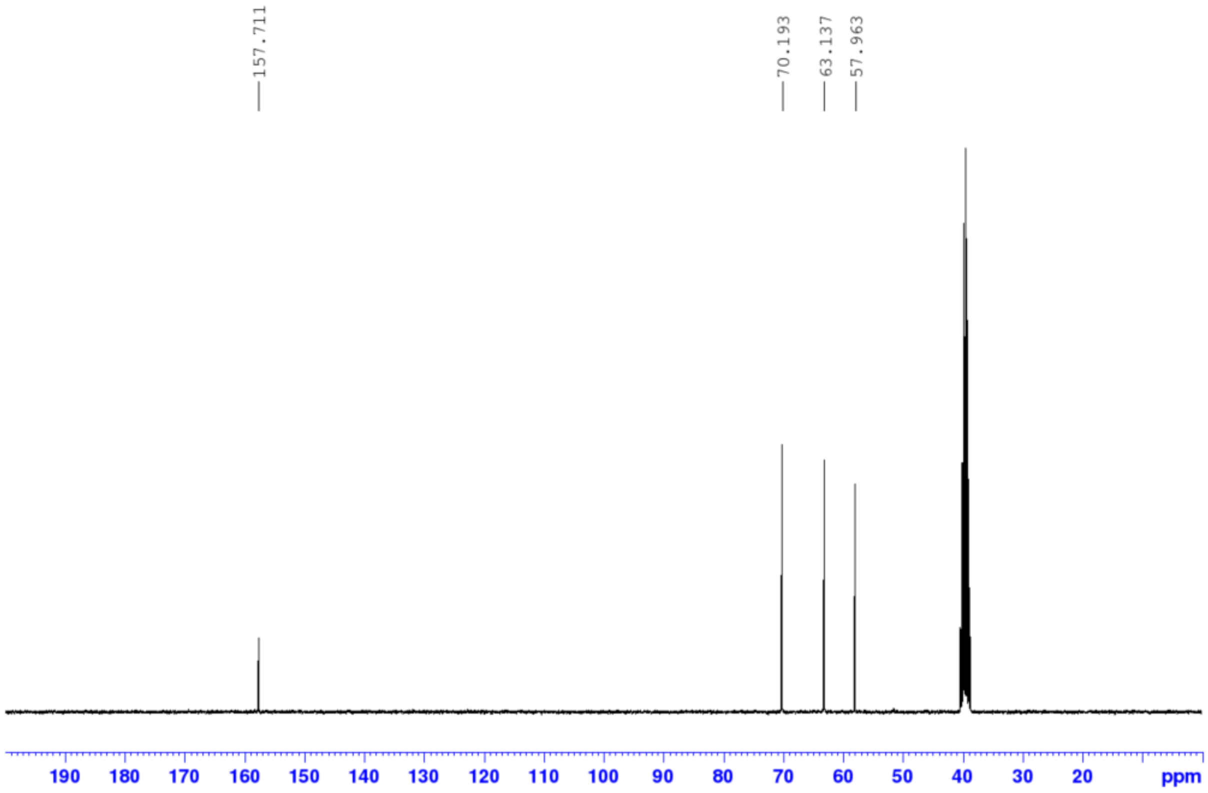

tetrahydropyran-4-yl N-hydroxycarbamate (**3k**)

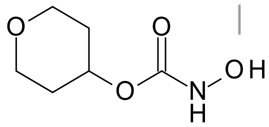

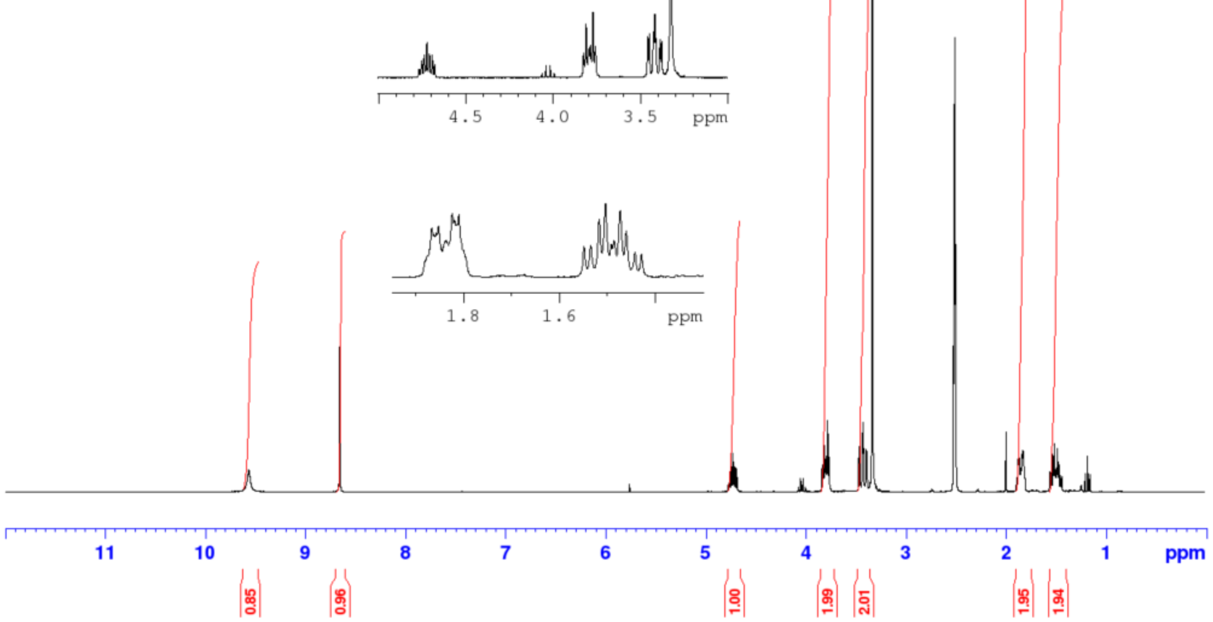

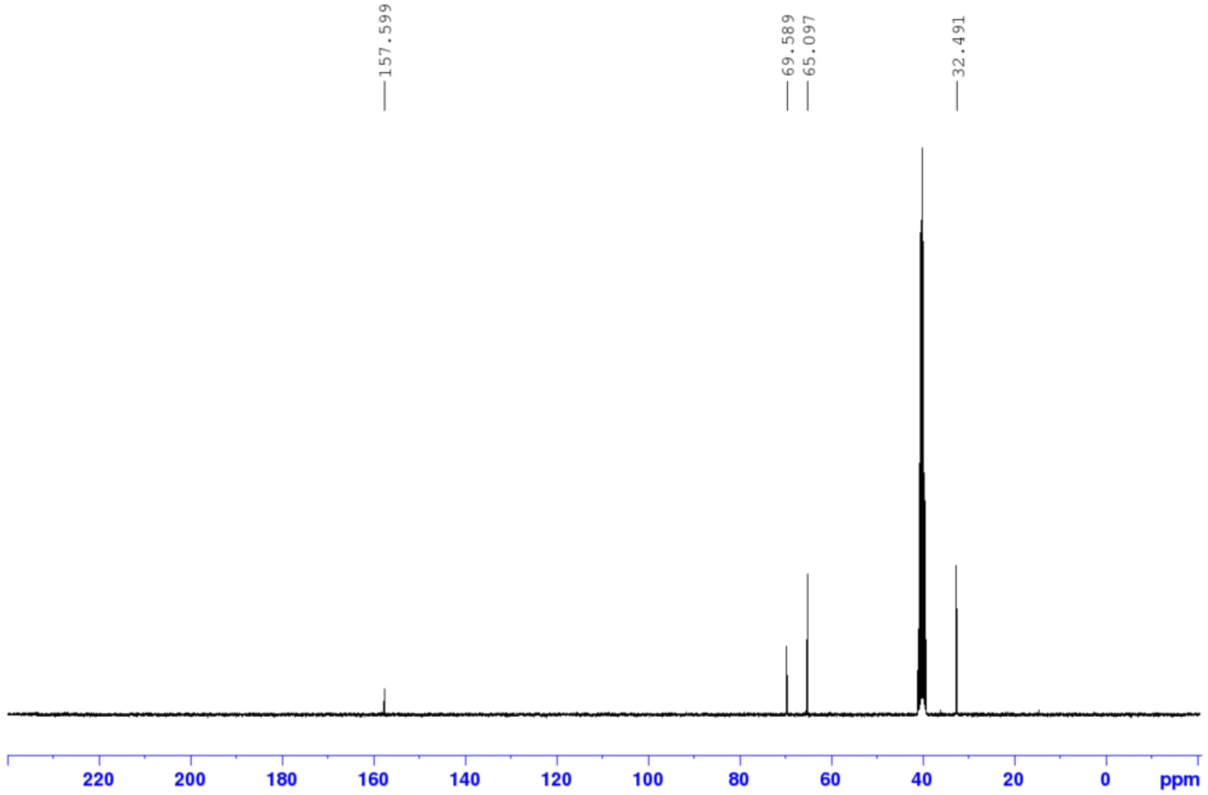

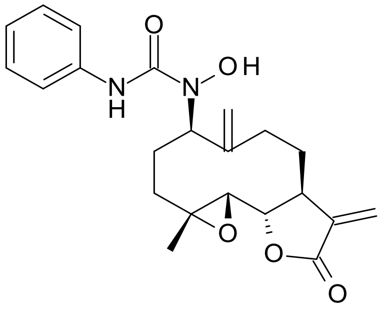

1-hydroxy-1-[(1S,2S,4R,7R,11S)-4-methyl-8,12-dimethylene-13-oxo-3,14-dioxatricyclo[9.3.0.02,4] tetradecan-7-yl]-3-phenyl-urea (**4**)

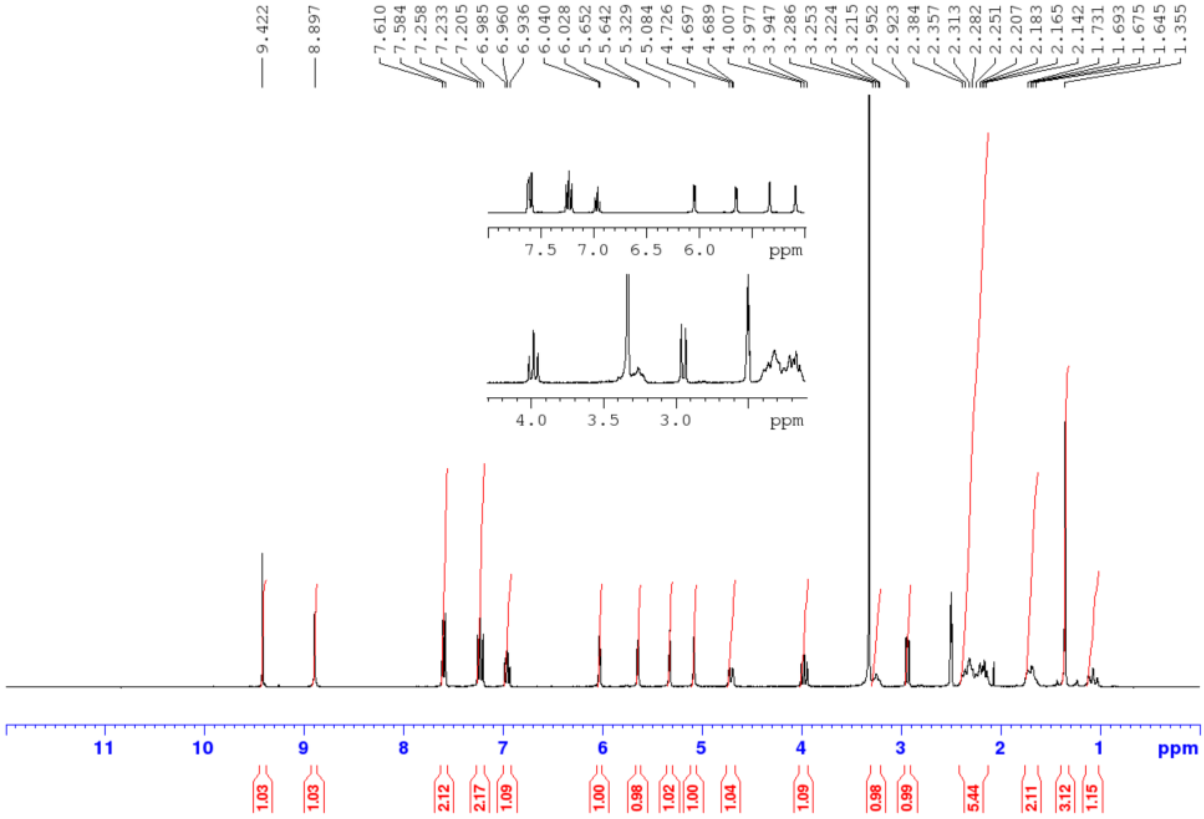

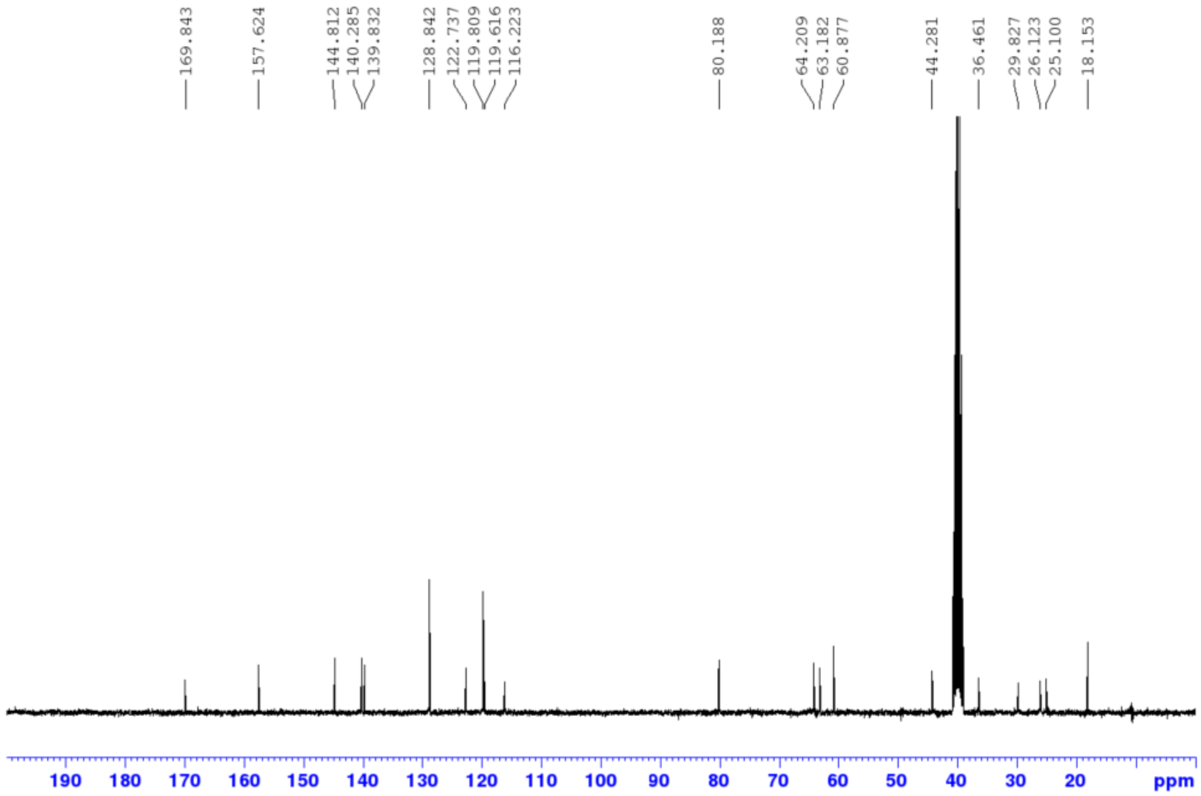

169.843
157.624
144.812
140.285
139.832
128.842
122.737
119.809
119.616
116.223
80.188
64.209
63.182
60.877
44.281
36.461
29.827
26.123
25.100
18.153
190 180 170 160 150 140 130 120 110 100 90 80 70 60 50 40 30 20 ppm

1-hydroxy-1-[(1S,2S,4R,7R,11S)-4-methyl-8,12-dimethylene-13-oxo-3,14-dioxatricyclo[9.3.0.02,4] tetradecan-7-yl]-3-(p-tolyl)urea (**5**)

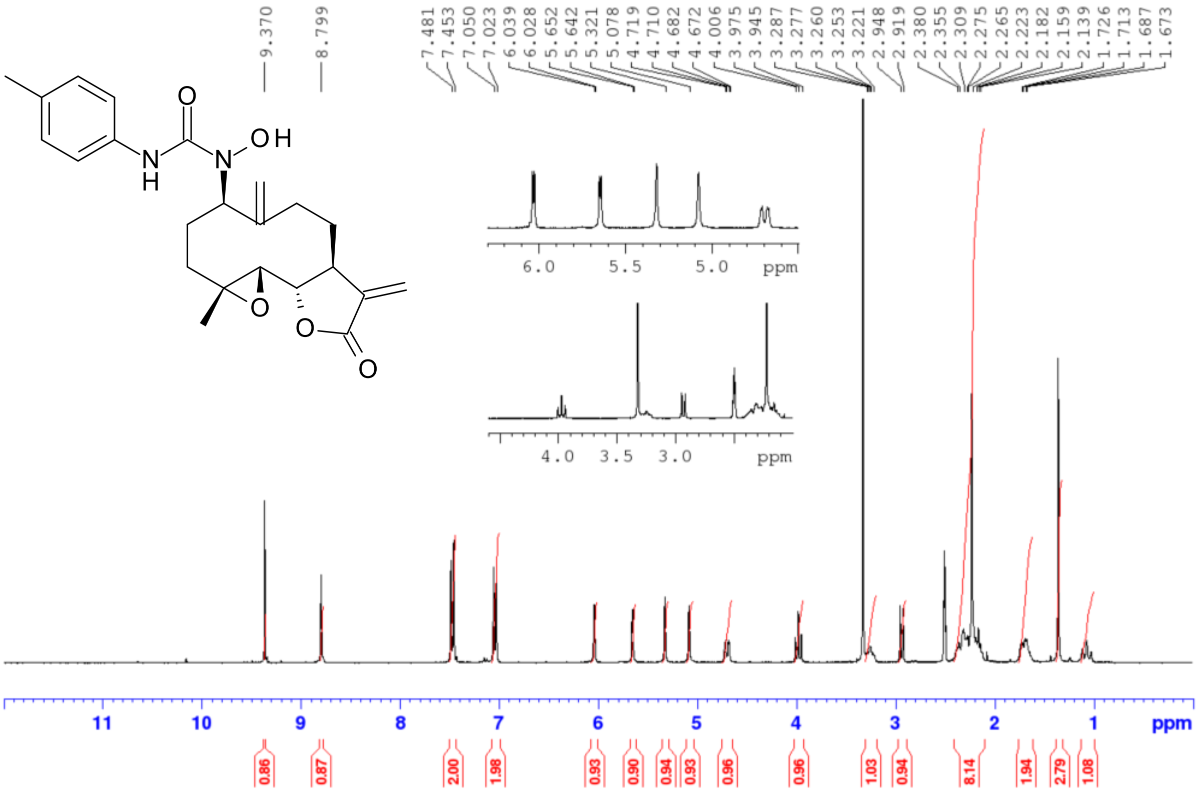

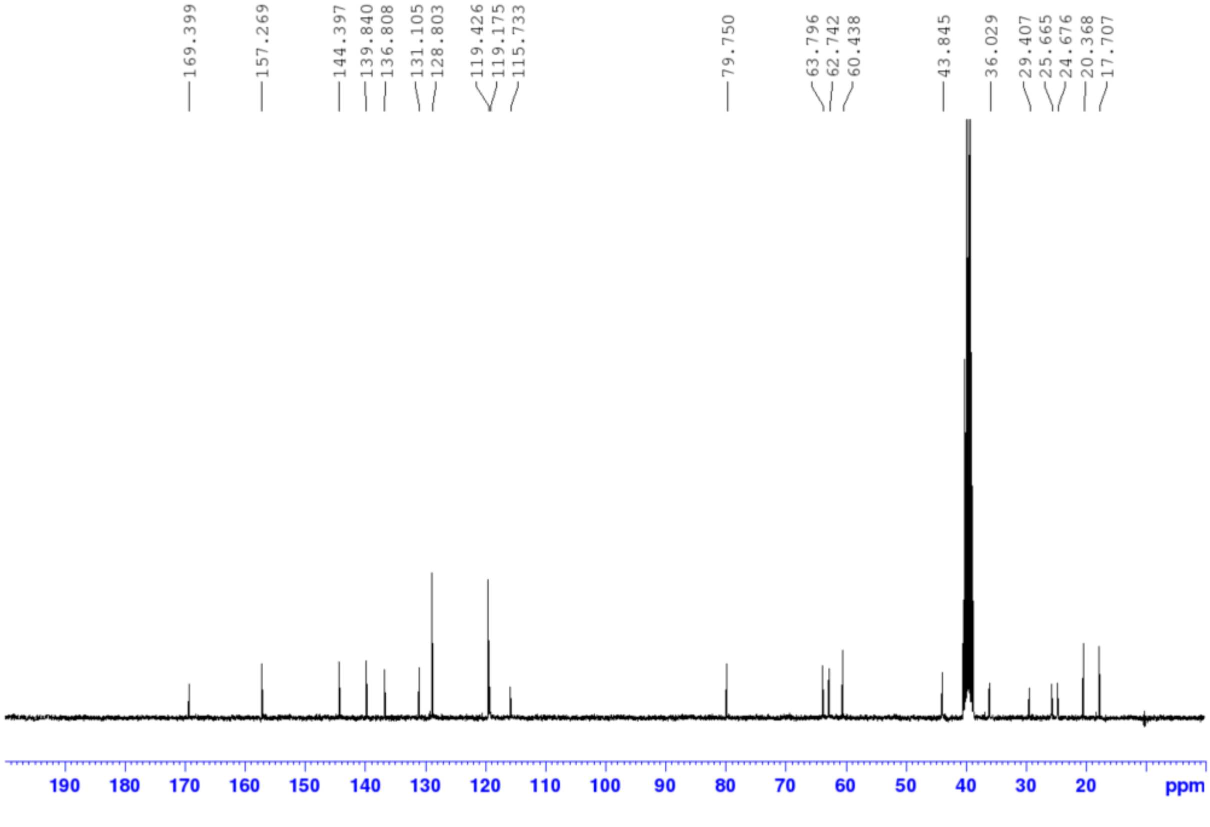

3-benzyl-1-hydroxy-1-[(1S,2S,4R,7R,11S)-4-methyl-8,12-dimethylene-13-oxo-3,14-dioxatricyclo [9.3.0.02,4]tetradecan-7-yl]urea (**6**)

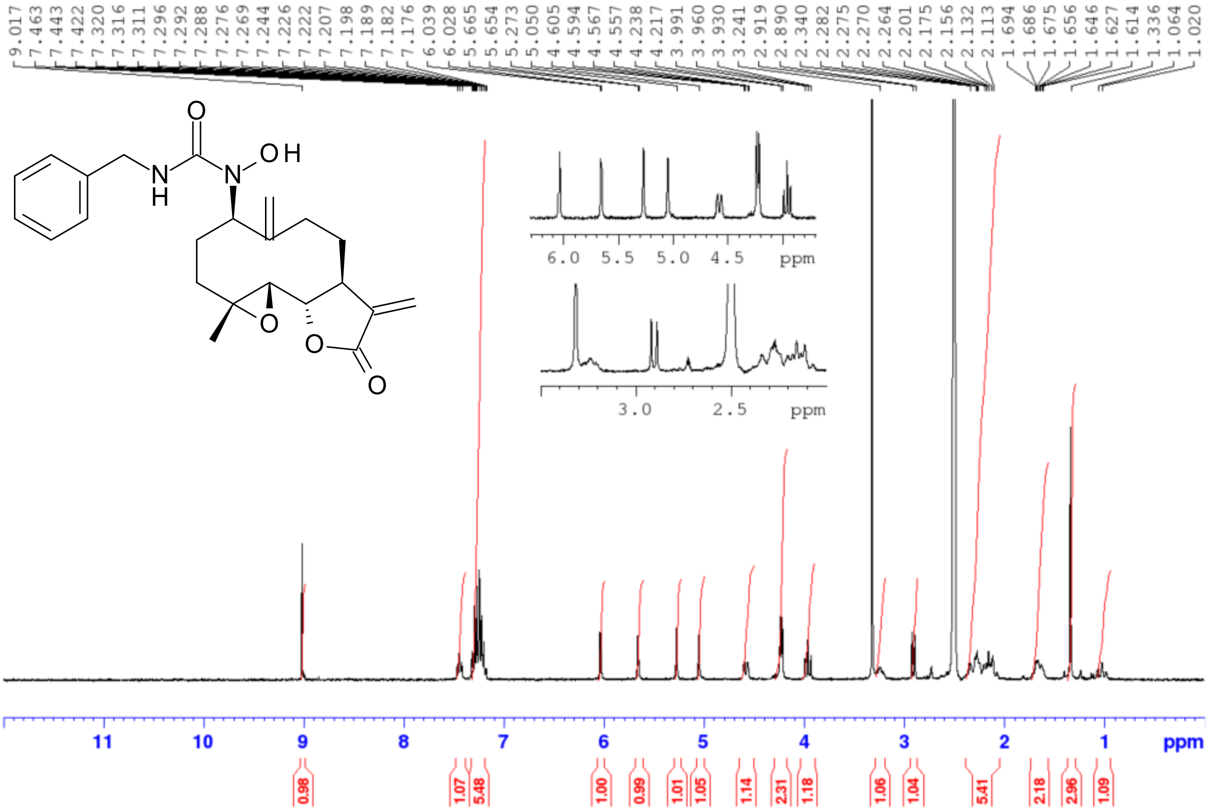

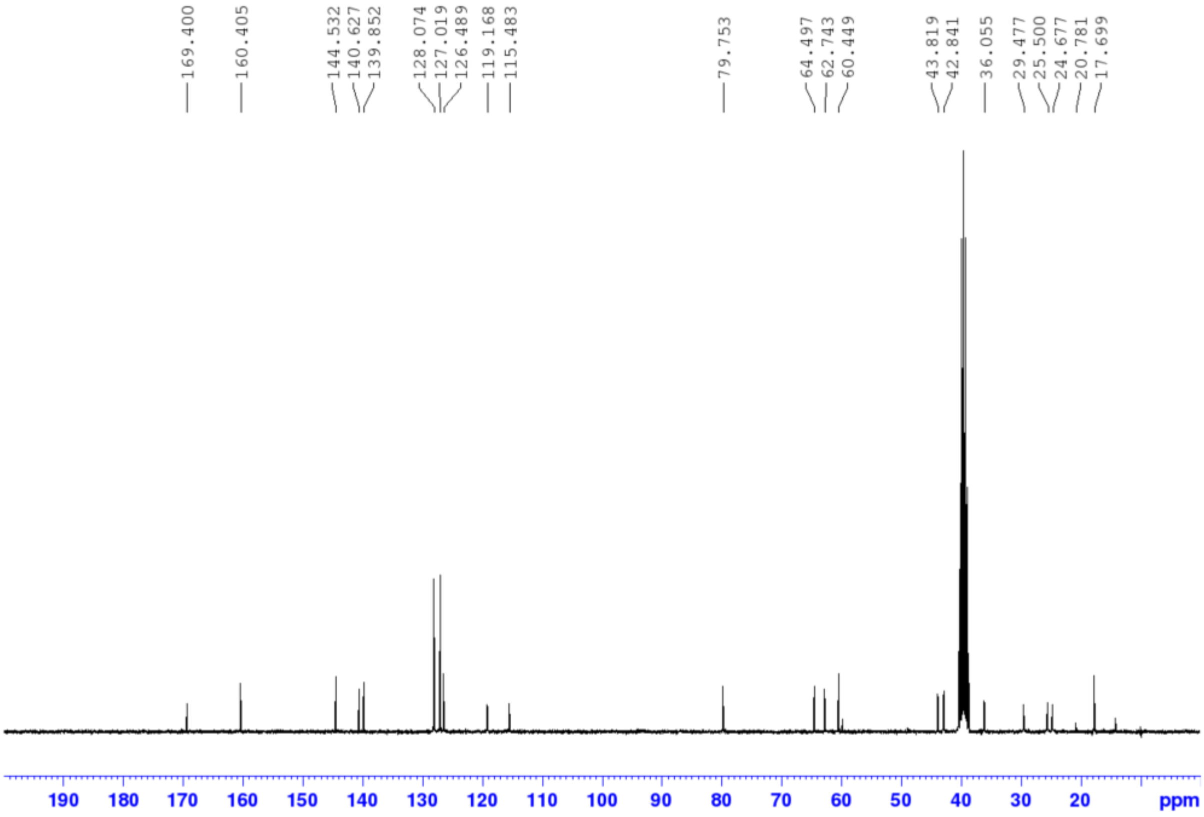

1-hydroxy-3-(4-methoxyphenyl)-1-[(1S,2S,4R,7R,11S)-4-methyl-8,12-dimethylene-13-oxo-3,14-dioxatricyclo[9.3.0.02,4]tetradecan-7-yl]urea (**7**)

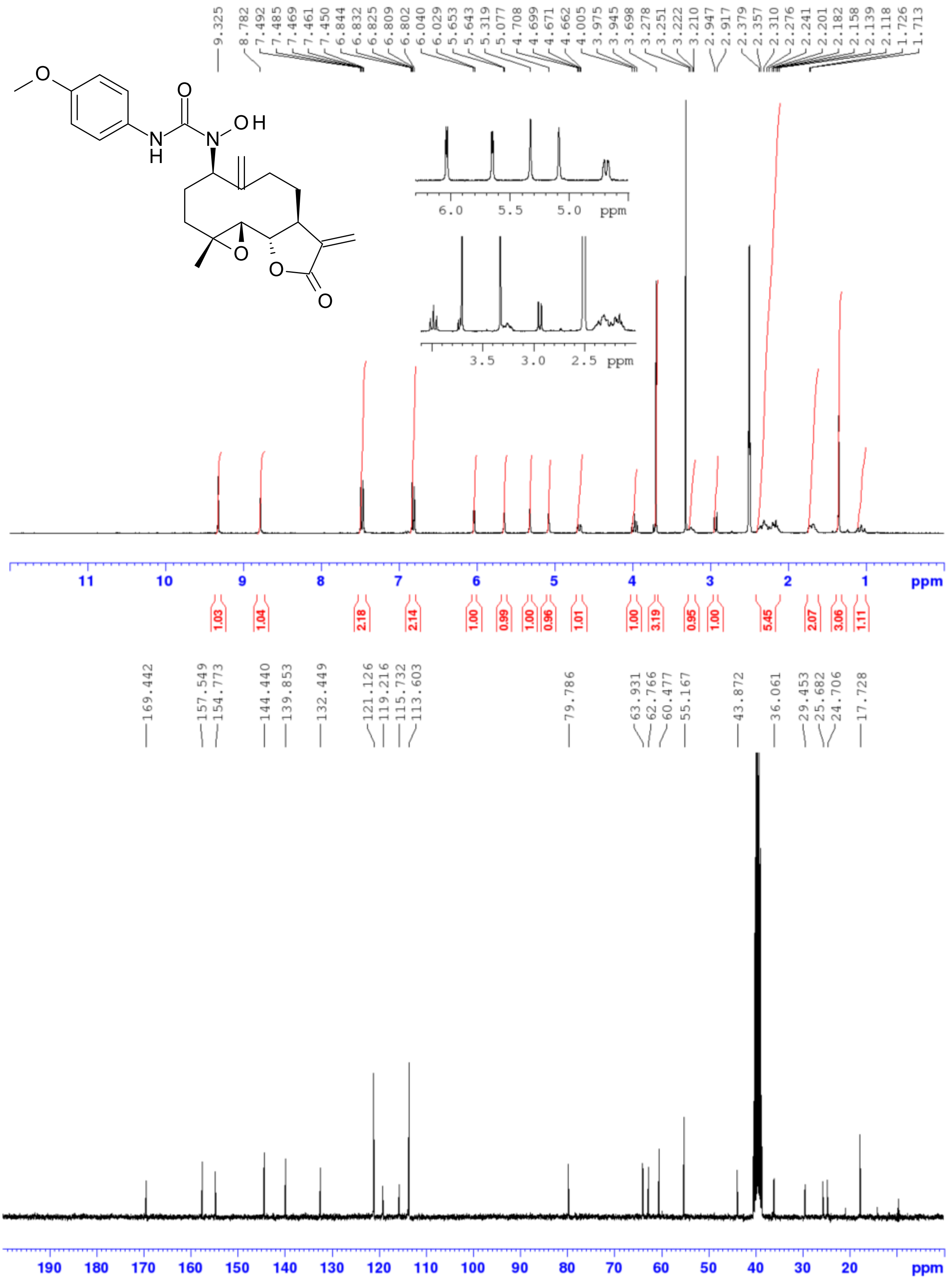

3-(4-fluorophenyl)-1-hydroxy-1-[(1S,2S,4R,7R,11S)-4-methyl-8,12-dimethylene-13-oxo-3,14-dioxatricyclo[9.3.0.02,4]tetradecan-7-yl]urea (**8**)

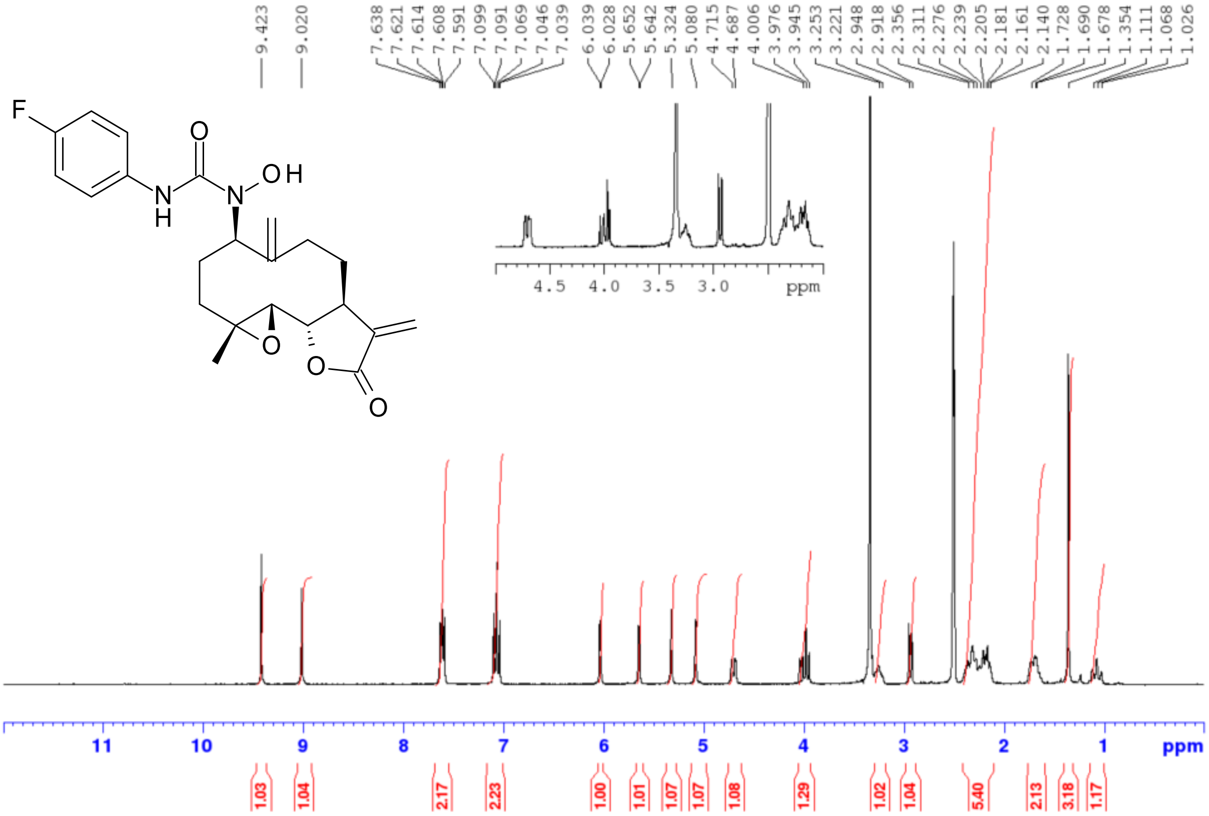

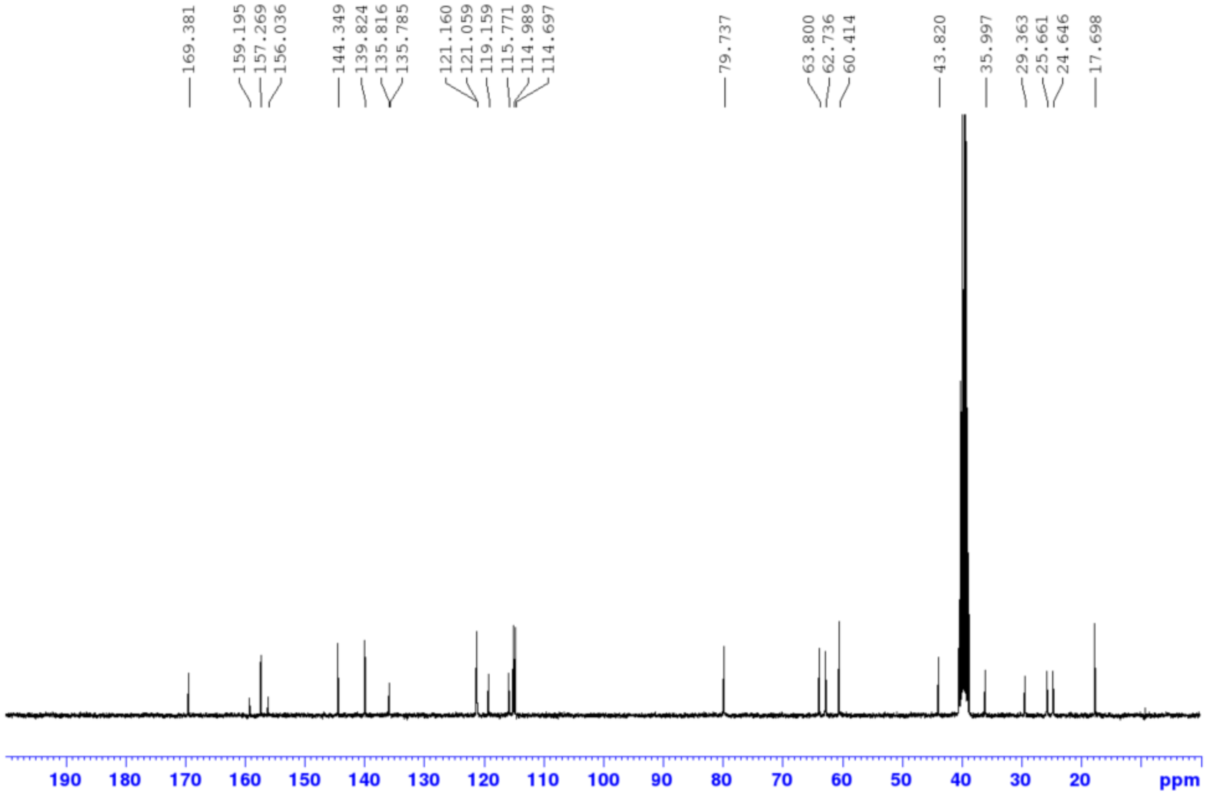

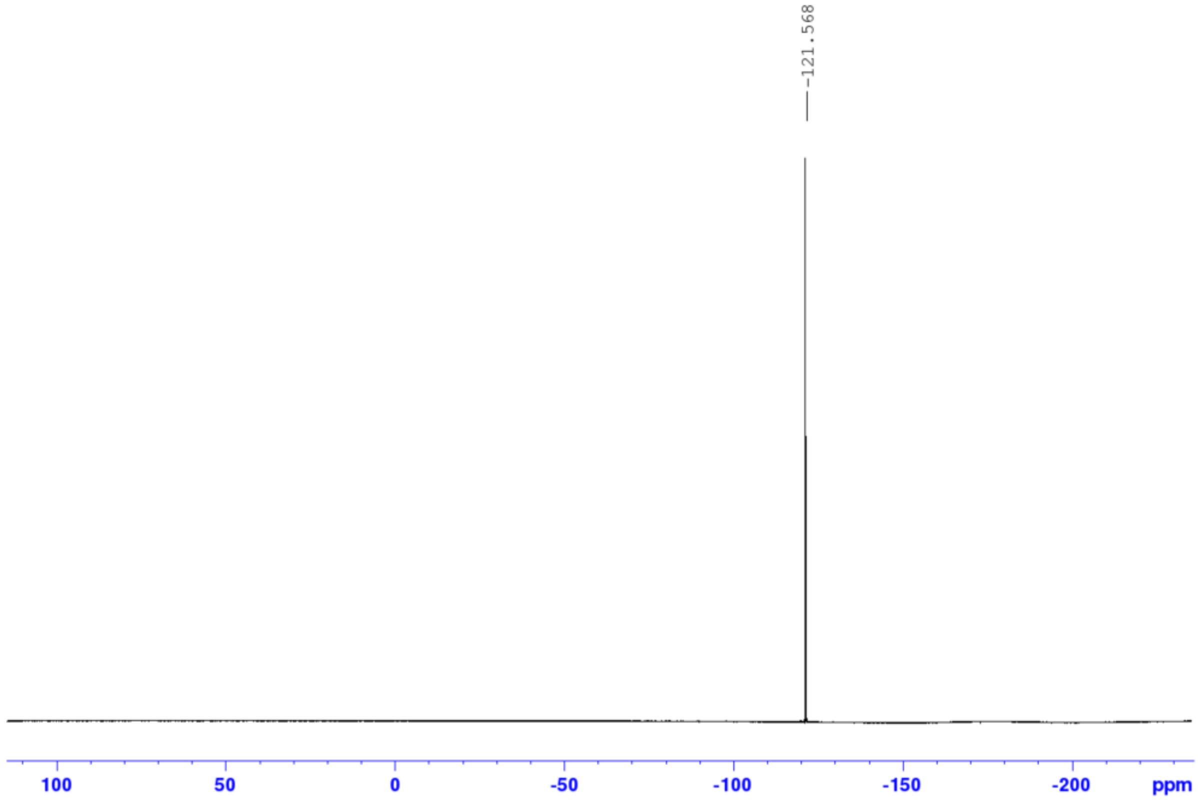
-121.568
100
50
0
-50
-100
-150
-200
ppm

1-hydroxy-1-[(1S,2S,4R,7R,11S)-4-methyl-8,12-dimethylene-13-oxo-3,14-dioxatricyclo[9.3.0.02,4]tetradecan-7-yl]urea (**9**)

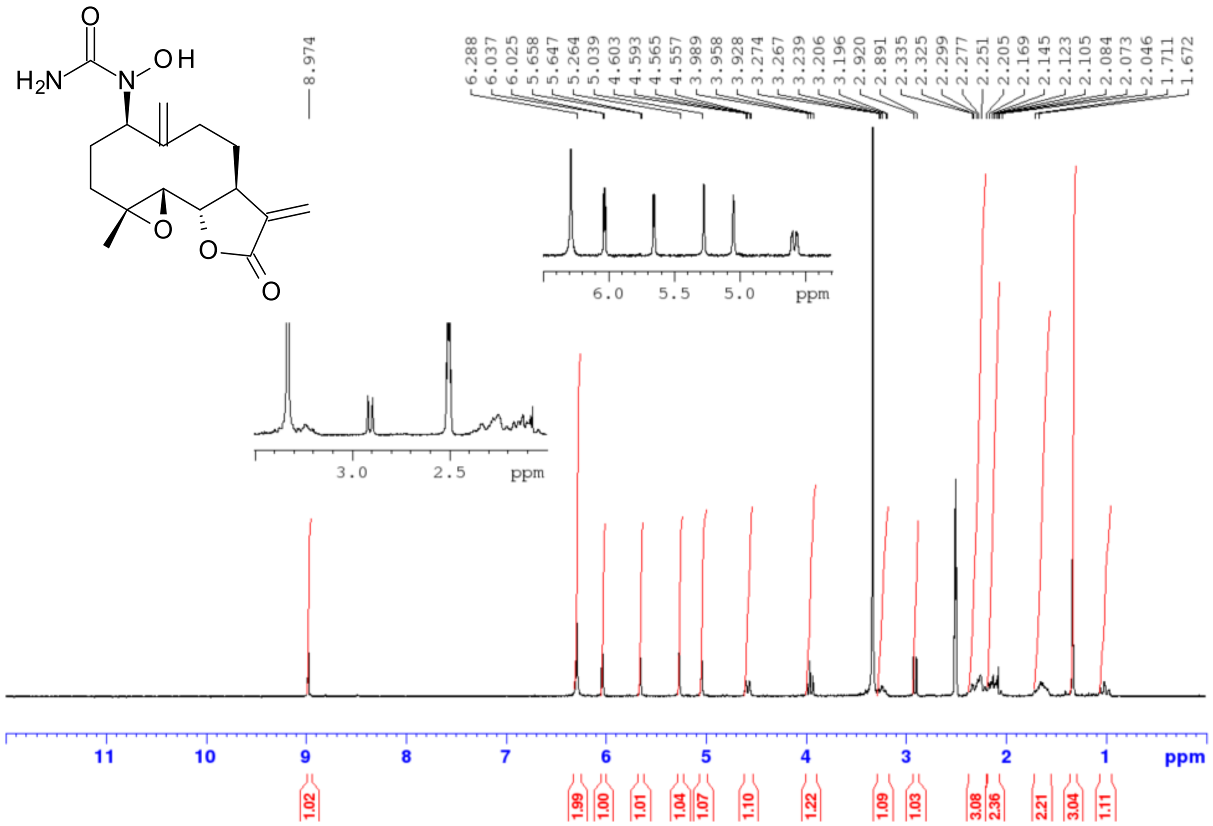

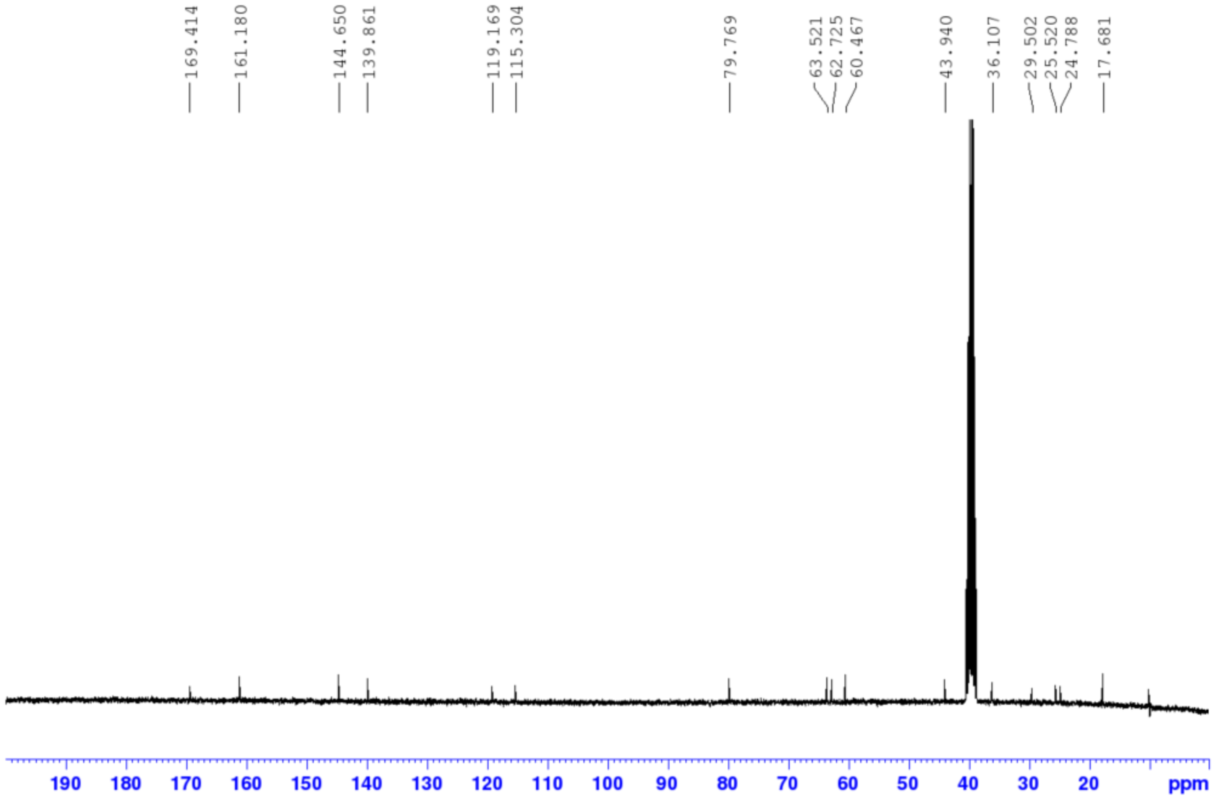

3-(1,3-benzodioxol-5-yl)-1-hydroxy-1-[(1S,2S,4R,7R,11S)-4-methyl-8,12-dimethylene-13-oxo-3,14-dioxatricyclo[9.3.0.02,4] tetradecan-7-yl]urea (**10**)

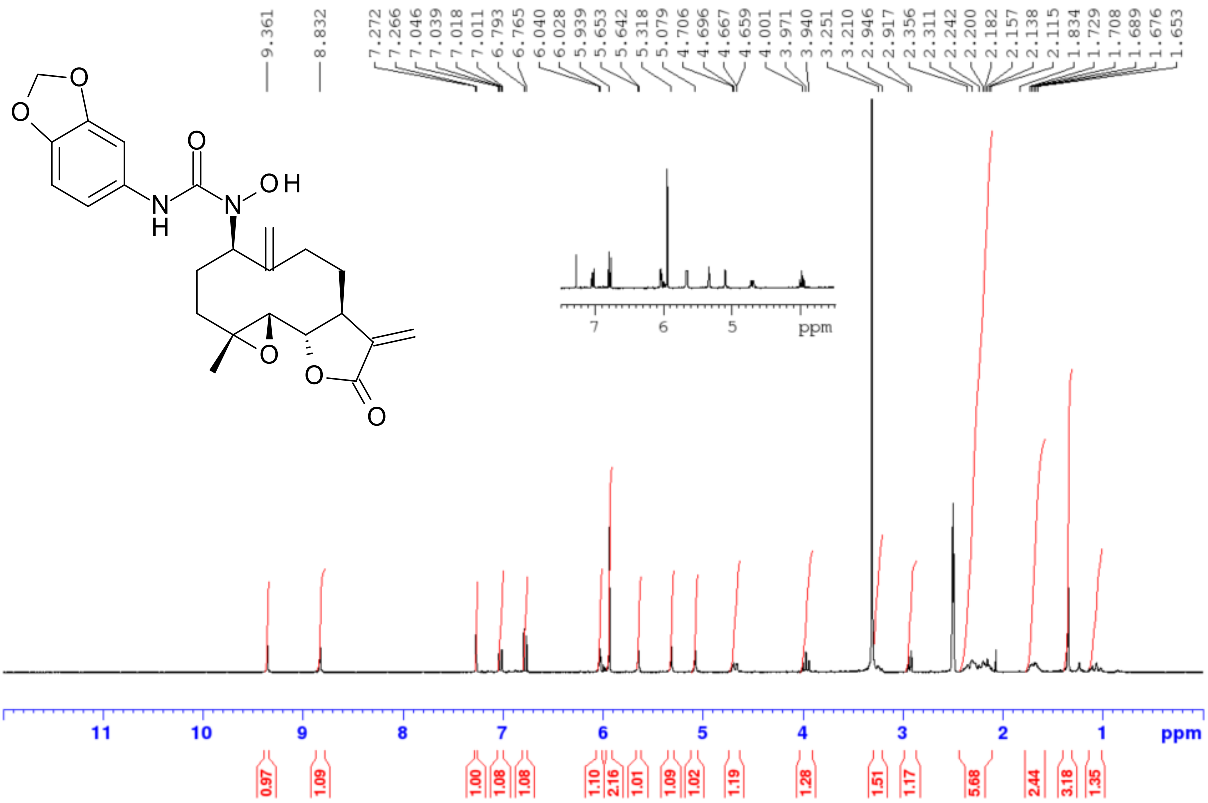

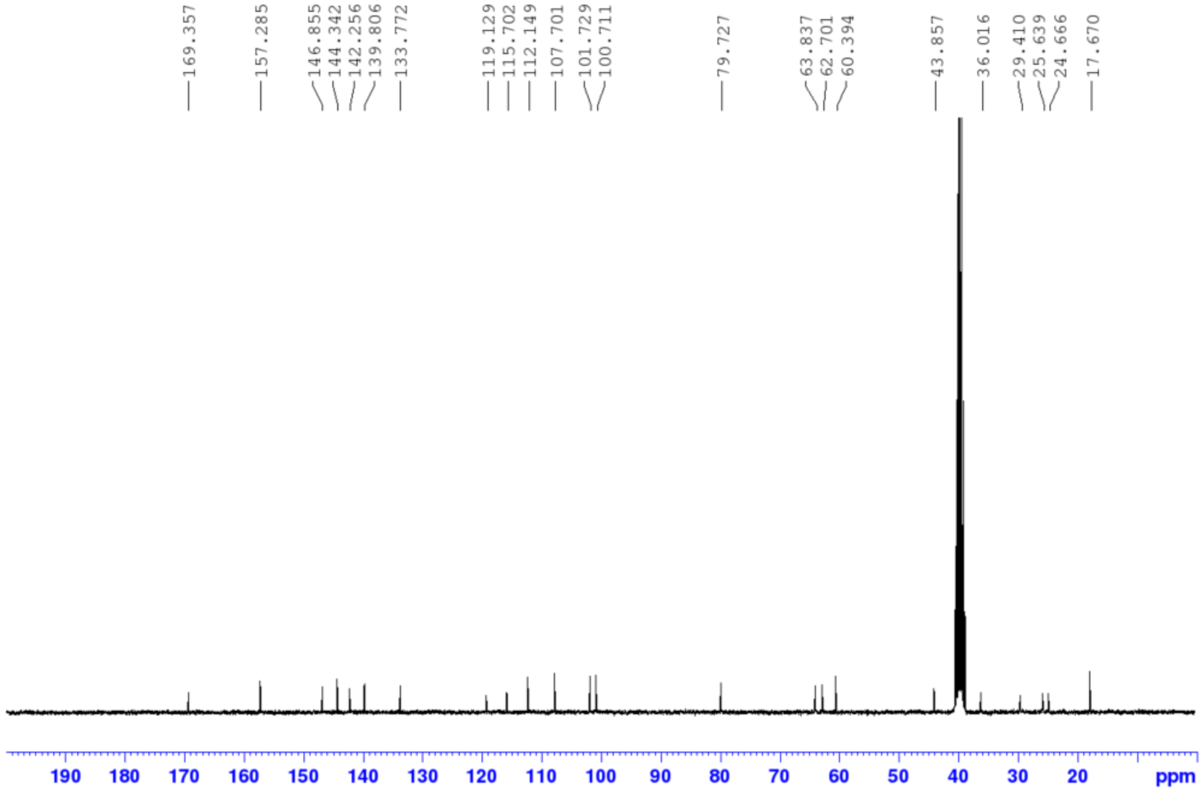

3-(3-ethoxypropyl)-1-hydroxy-1-[(1S,2S,4R,7R,11S)-4-methyl-8,12-dimethylene-13-oxo-3,14-dioxatricyclo[9.3.0.02,4]tetradecan-7-yl]urea (**11**)

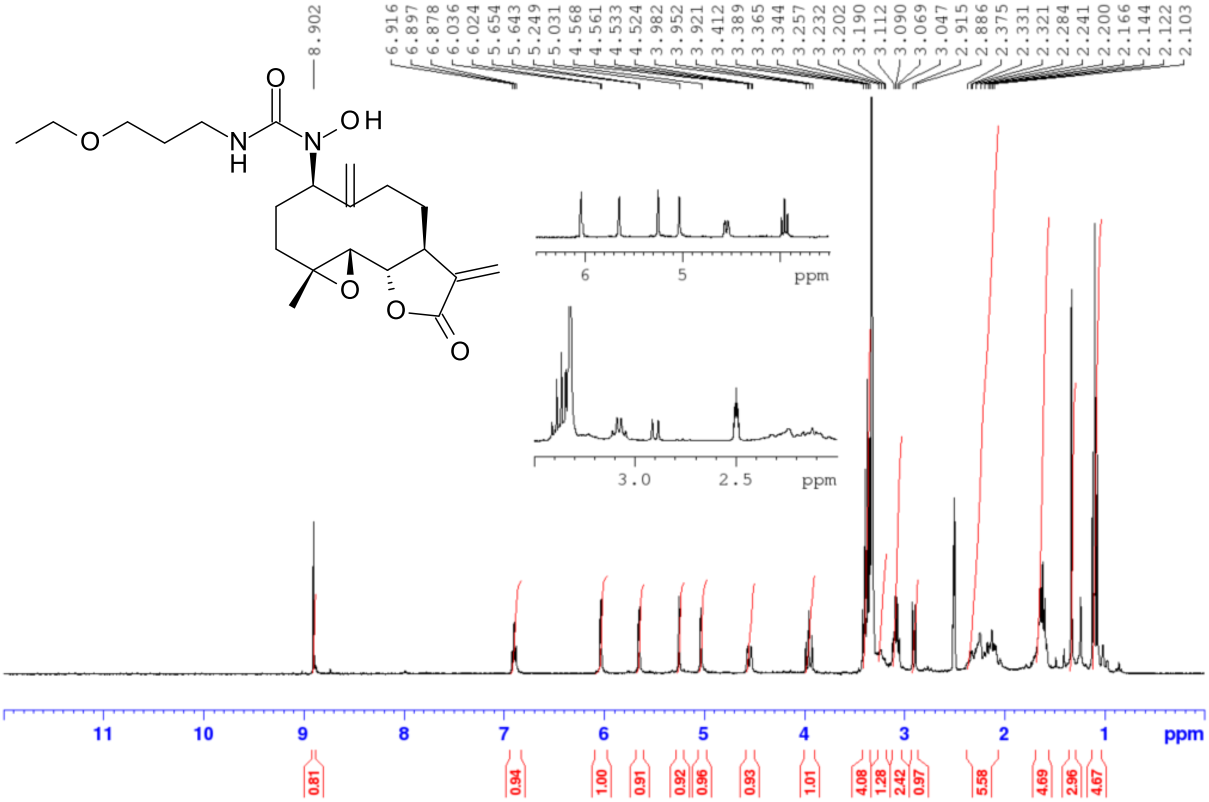

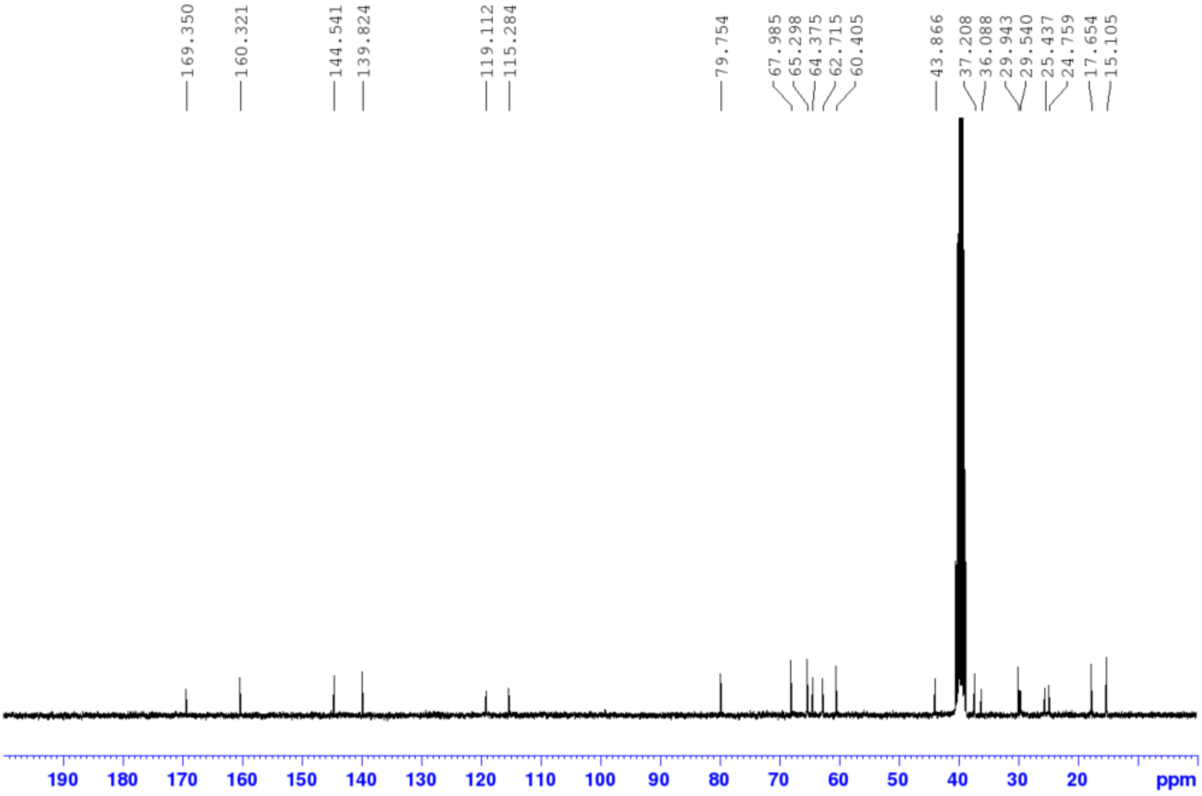

tert-butyl N-hydroxy-N-[(1S,2S,4R,7R,11S)-4-methyl-8,12-dimethylene-13-oxo-3,14-dioxatricyclo[9.3.0.02,4]tetradecan-7-yl]carbamate (**12**)

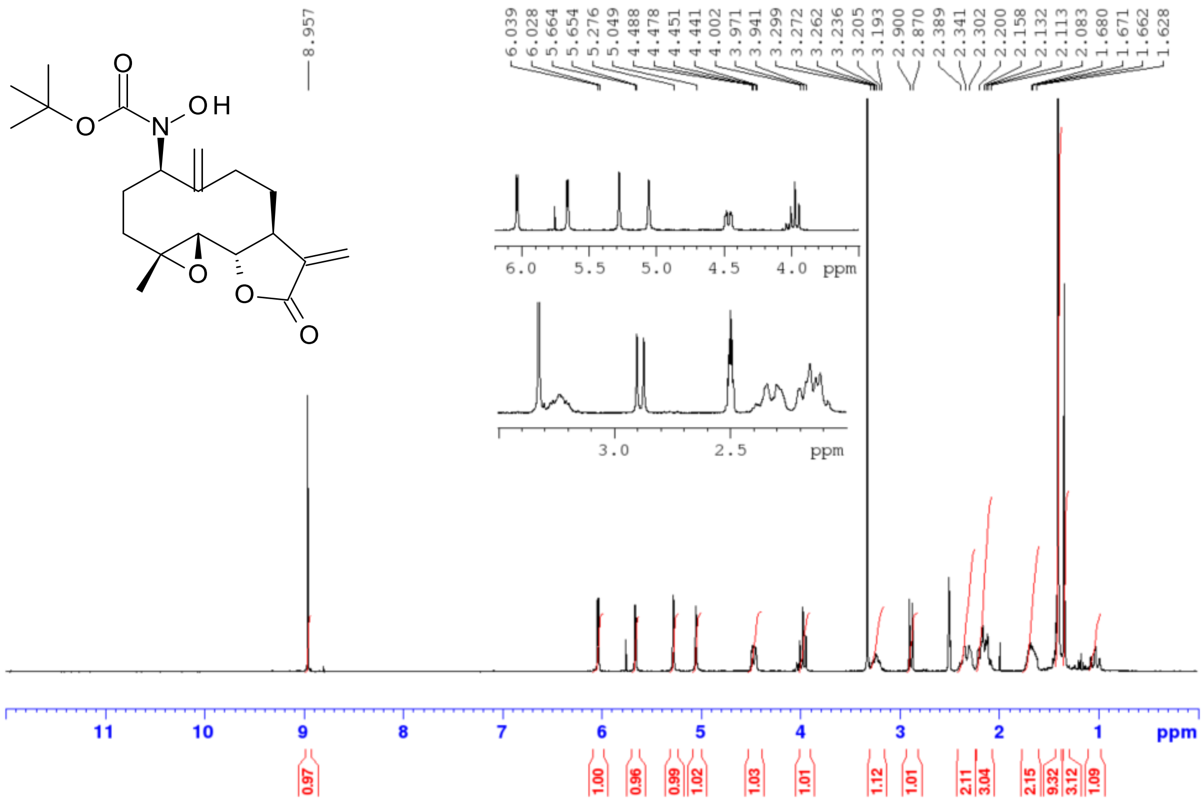

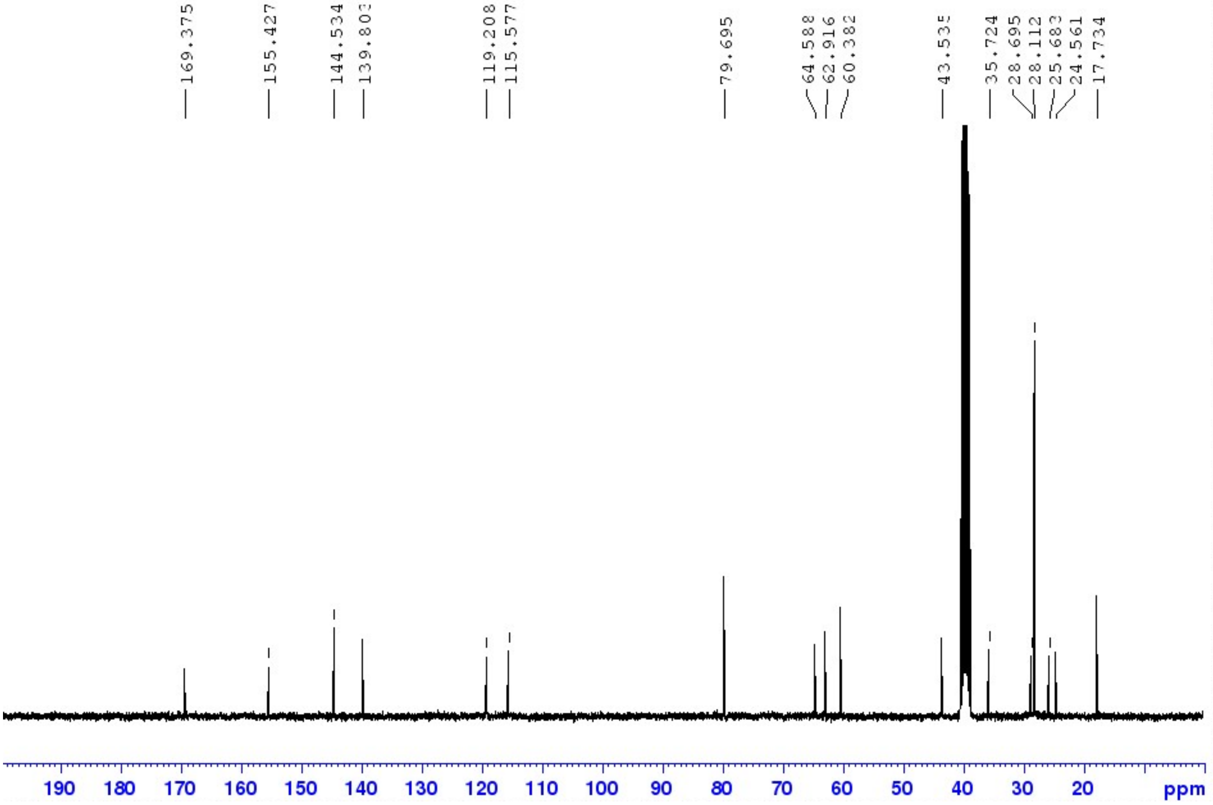

allyl N-hydroxy-N-[(1S,2S,4R,7R,11S)-4-methyl-8,12-dimethylene-13-oxo-3,14-dioxatricyclo [9.3.0.02,4]tetradecan-7-yl]carbamate (**13**)

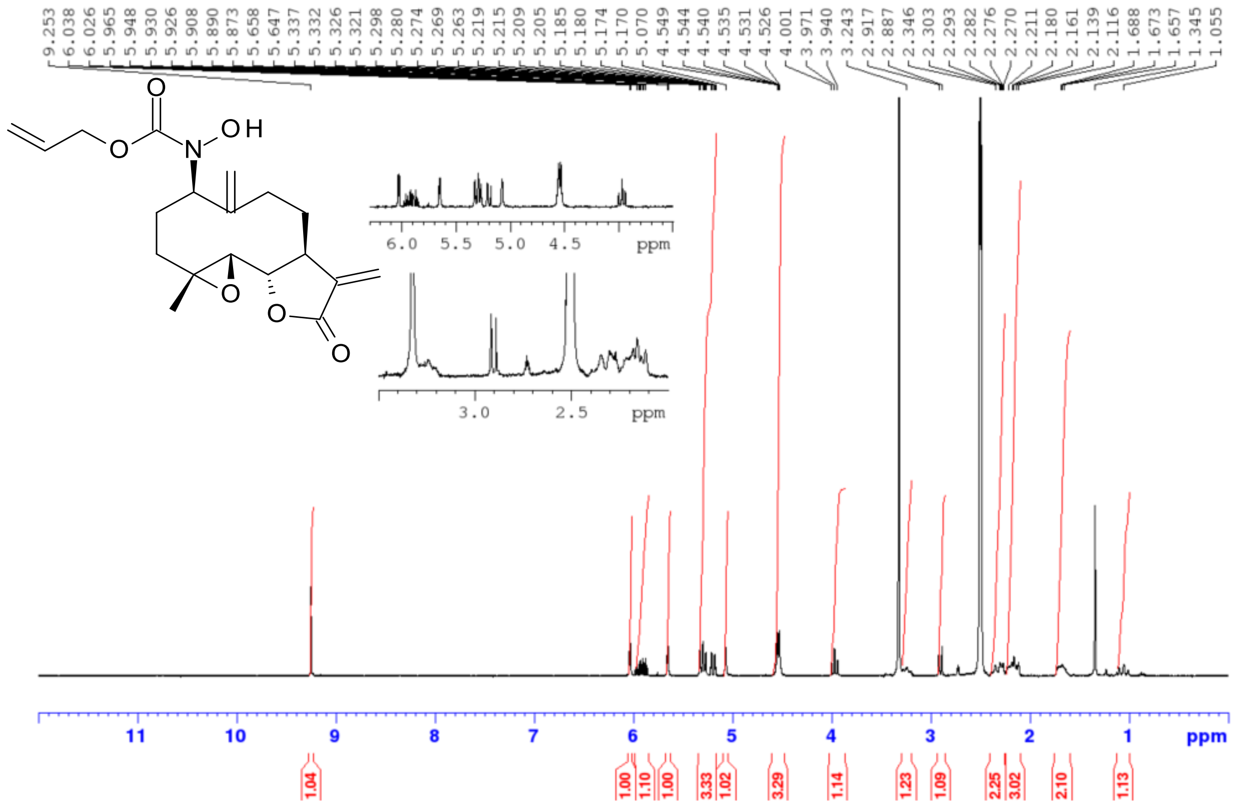

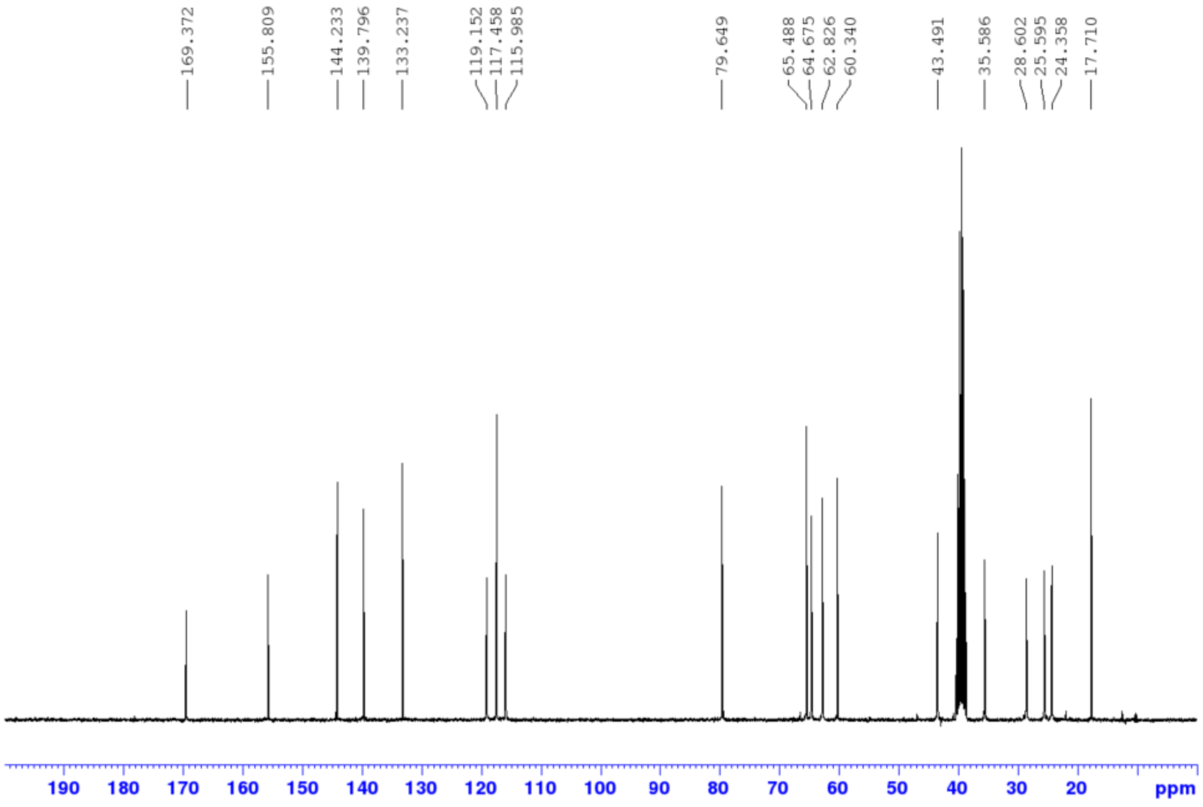

benzyl N-hydroxy-N-[(1S,2S,4R,7R,11S)-4-methyl-8,12-dimethylene-13-oxo-3,14-dioxatricyclo [9.3.0.02,4]tetradecan-7-yl]carbamate (**14**)

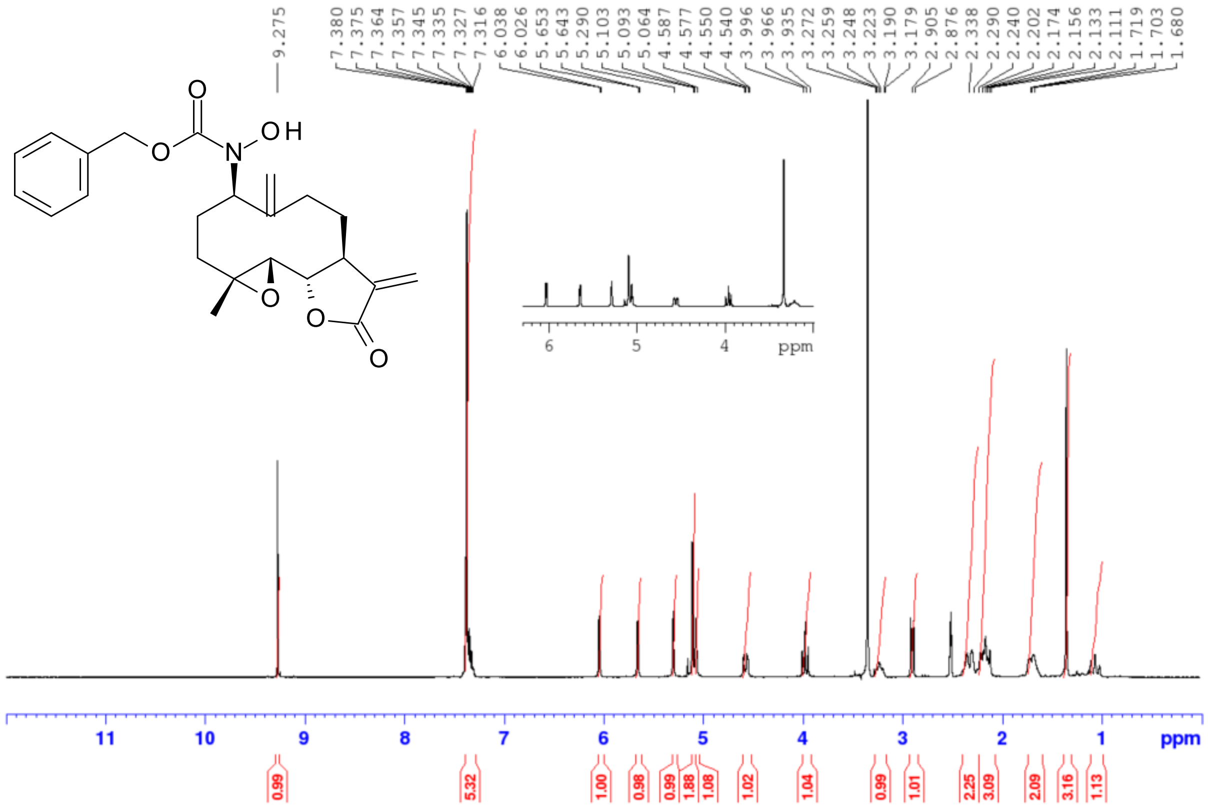

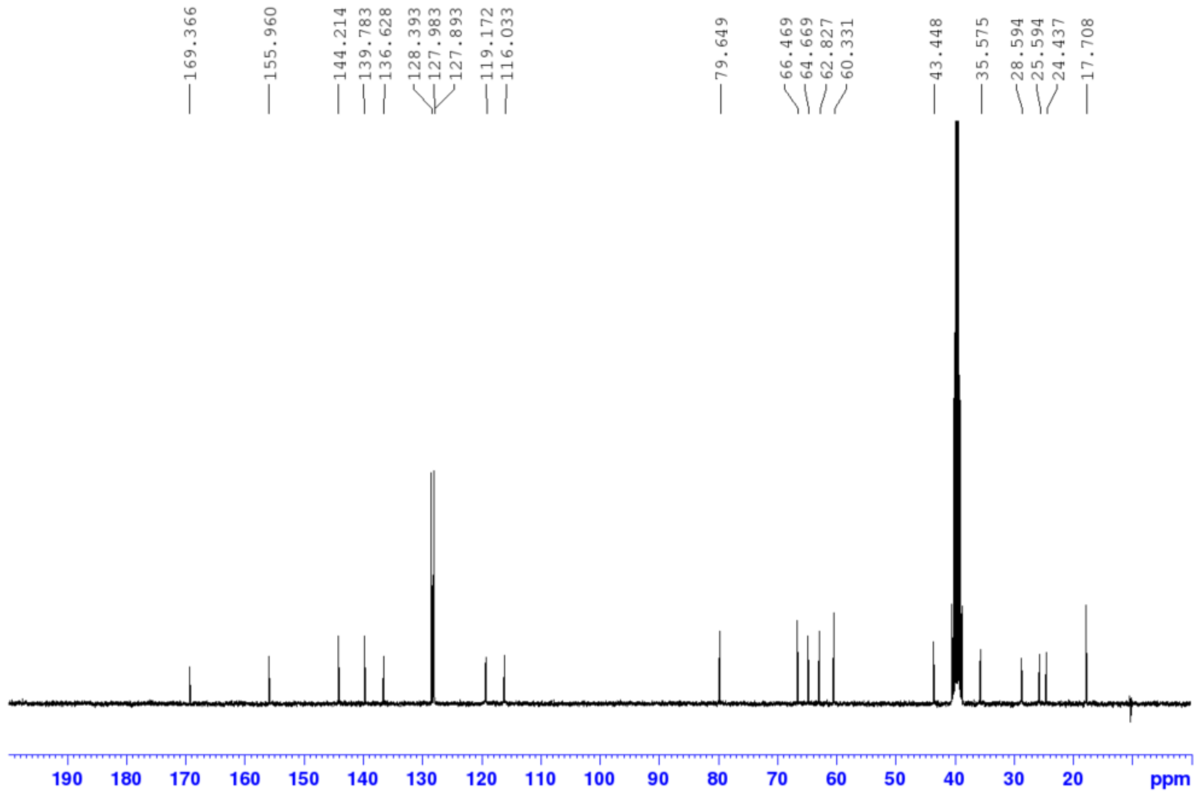

2,2,2-trichloroethyl N-hydroxy-N-[(1S,2S,4R,7R,11S)-4-methyl-8,12-dimethylene-13-oxo-3,14-dioxatricyclo[9.3.0.02,4]tetradecan-7-yl]carbamate (**15**)

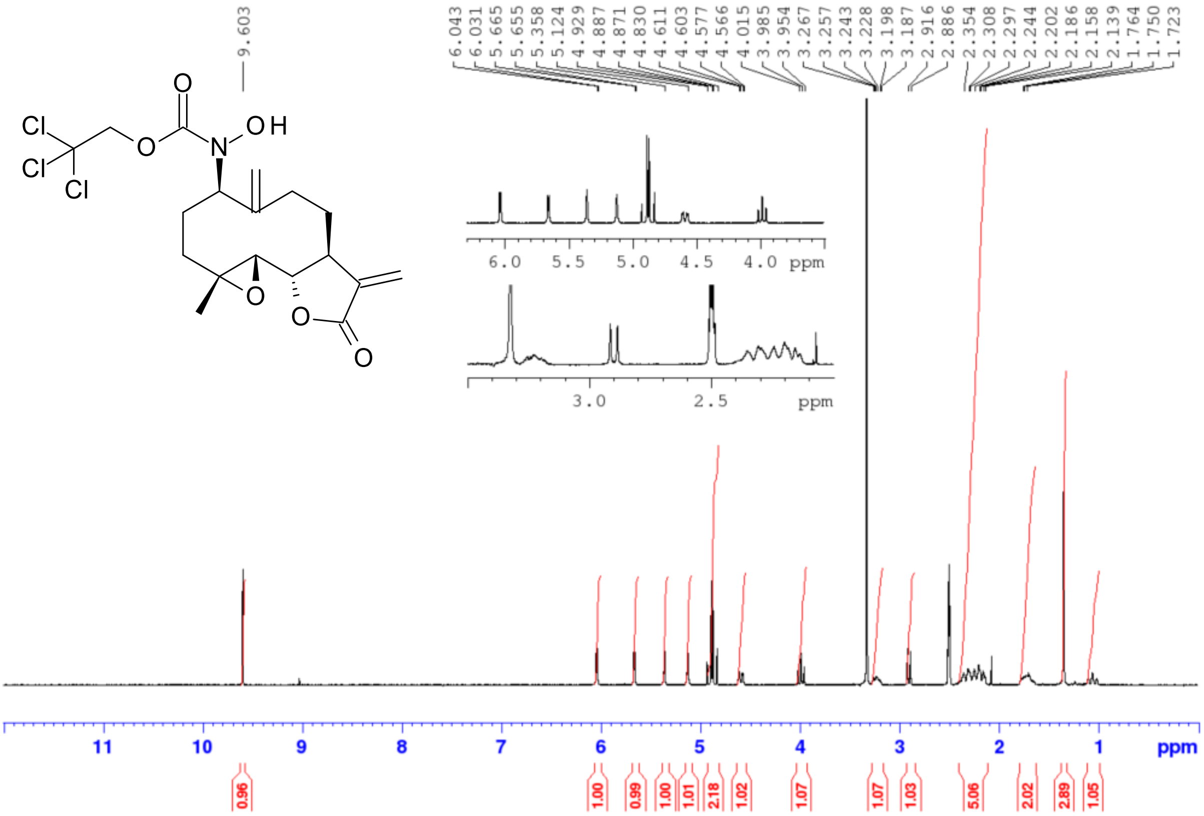

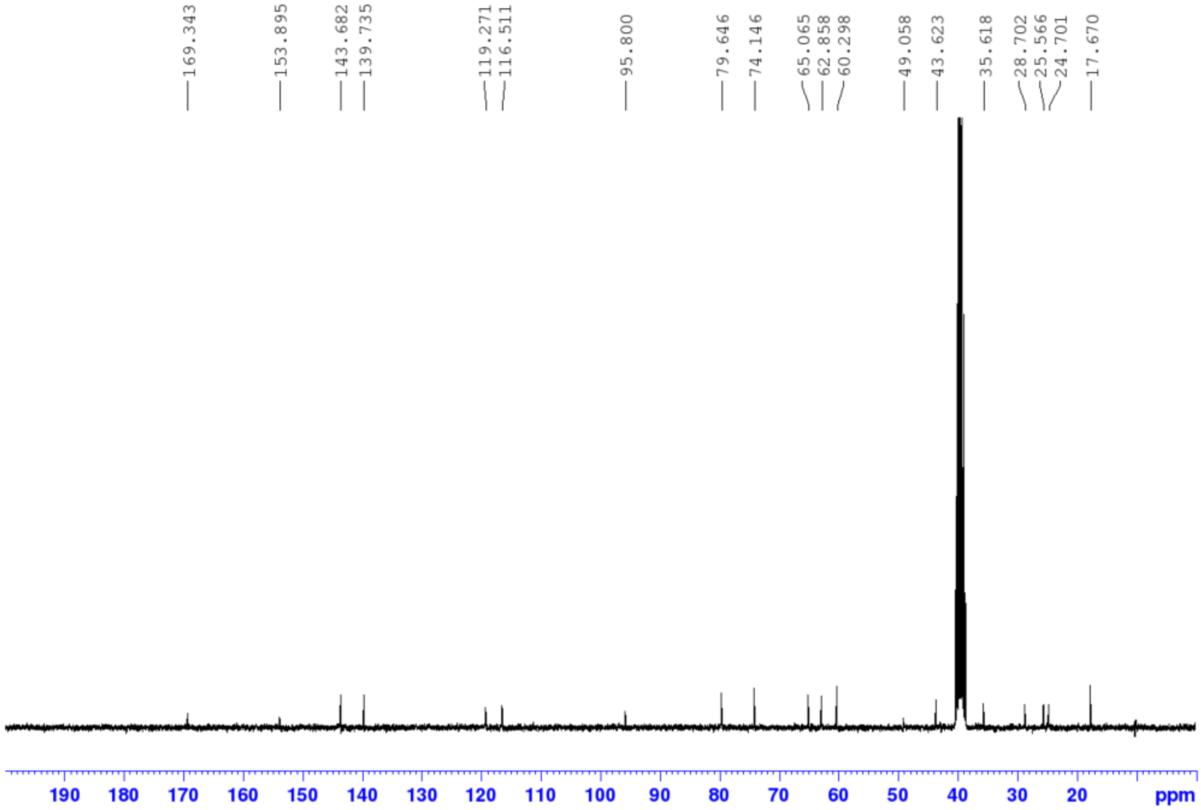

(4-fluorophenyl) N-hydroxy-N-[(1S,2S,4R,7R,11S)-4-methyl-8,12-dimethylene-13-oxo-3,14-dioxatricyclo[9.3.0.02,4]tetradecan-7-yl]carbamate (**16**)

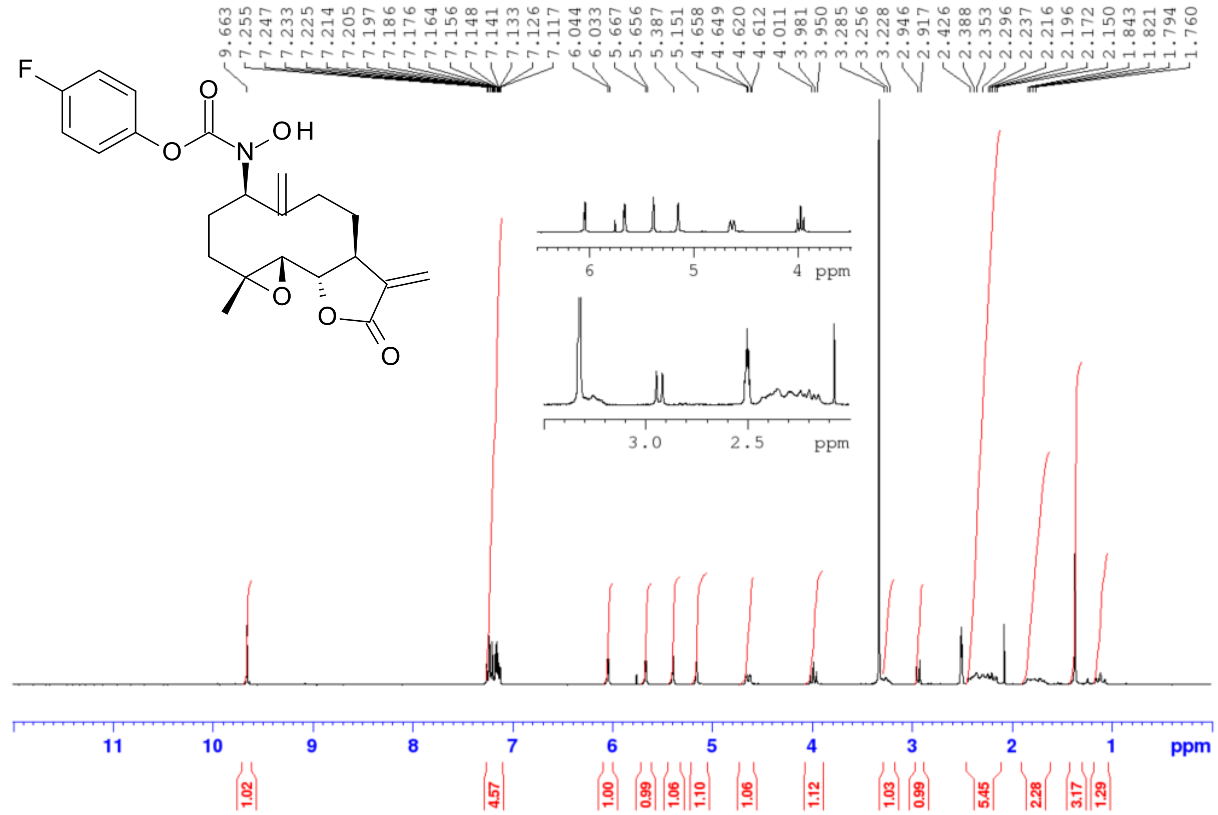

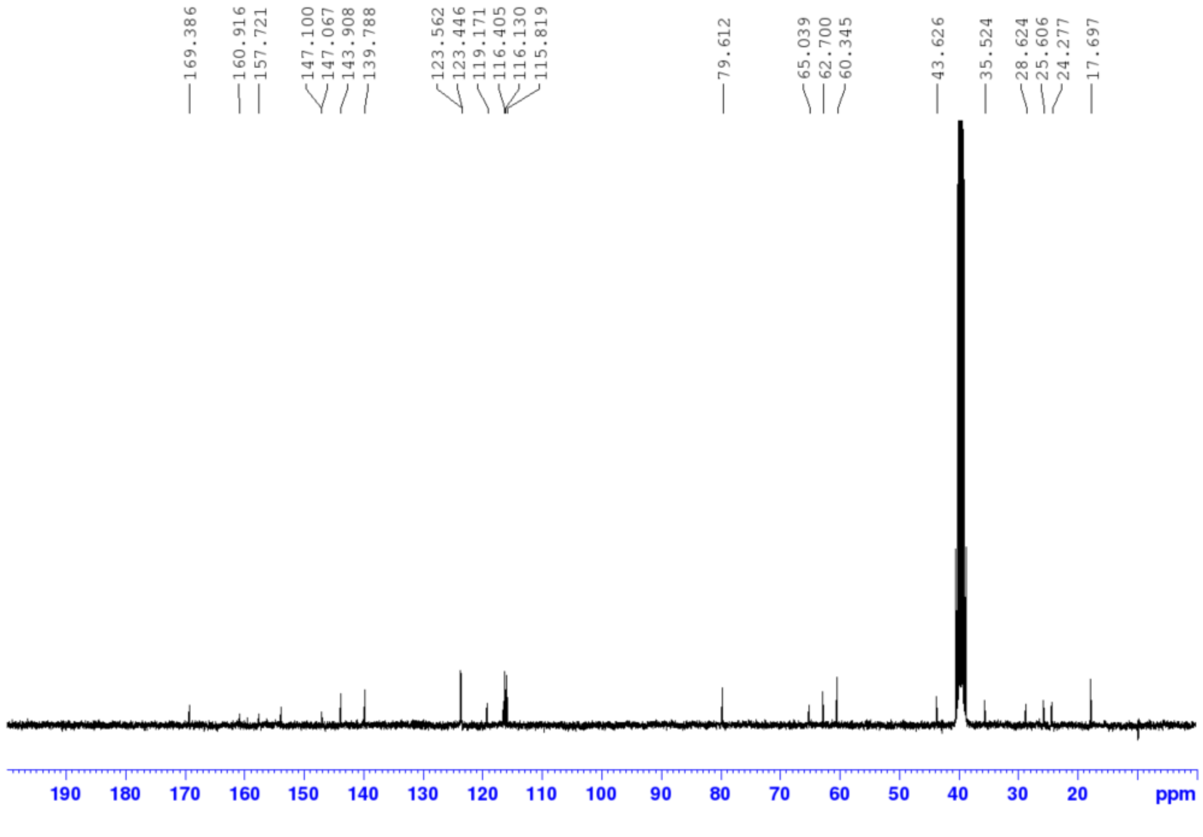

phenyl N-hydroxy-N-[(1S,2S,4R,7R,11S)-4-methyl-8,12-dimethylene-13-oxo-3,14-dioxatricyclo[9.3.0.02,4]tetradecan-7-yl]carbamate (**17**)

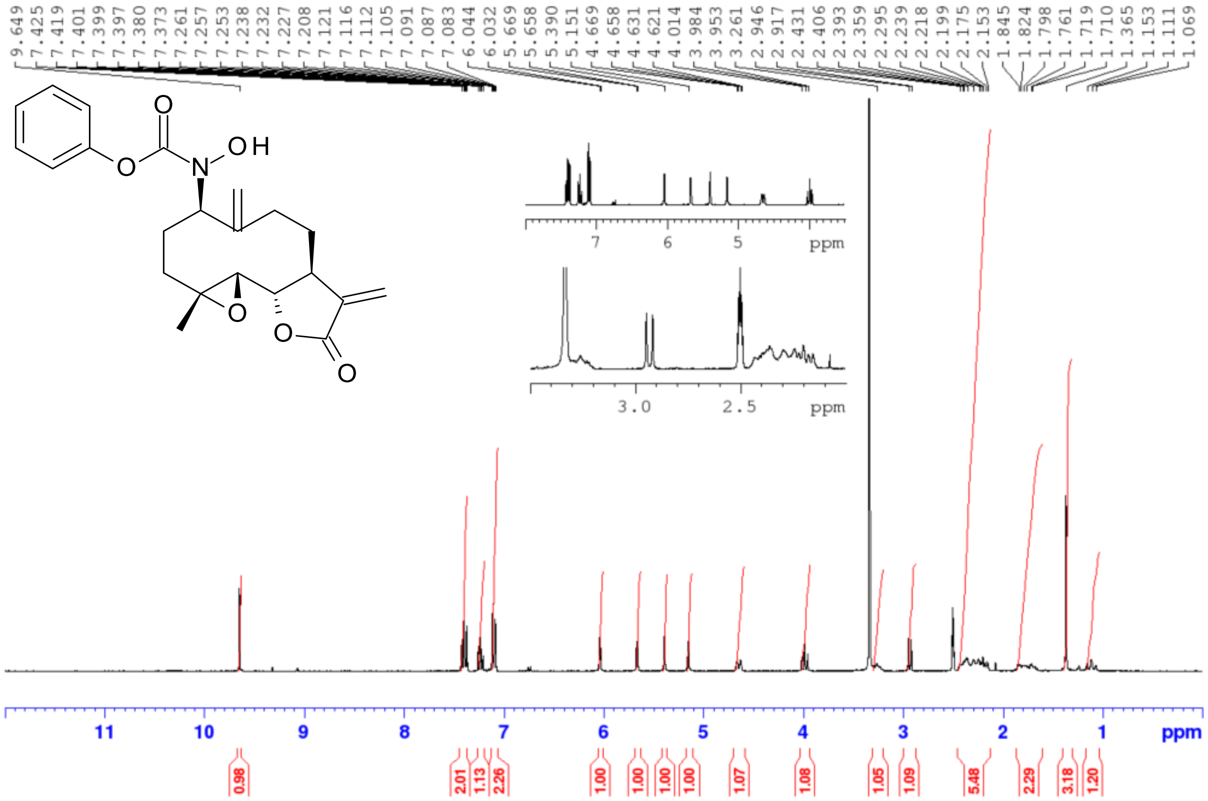

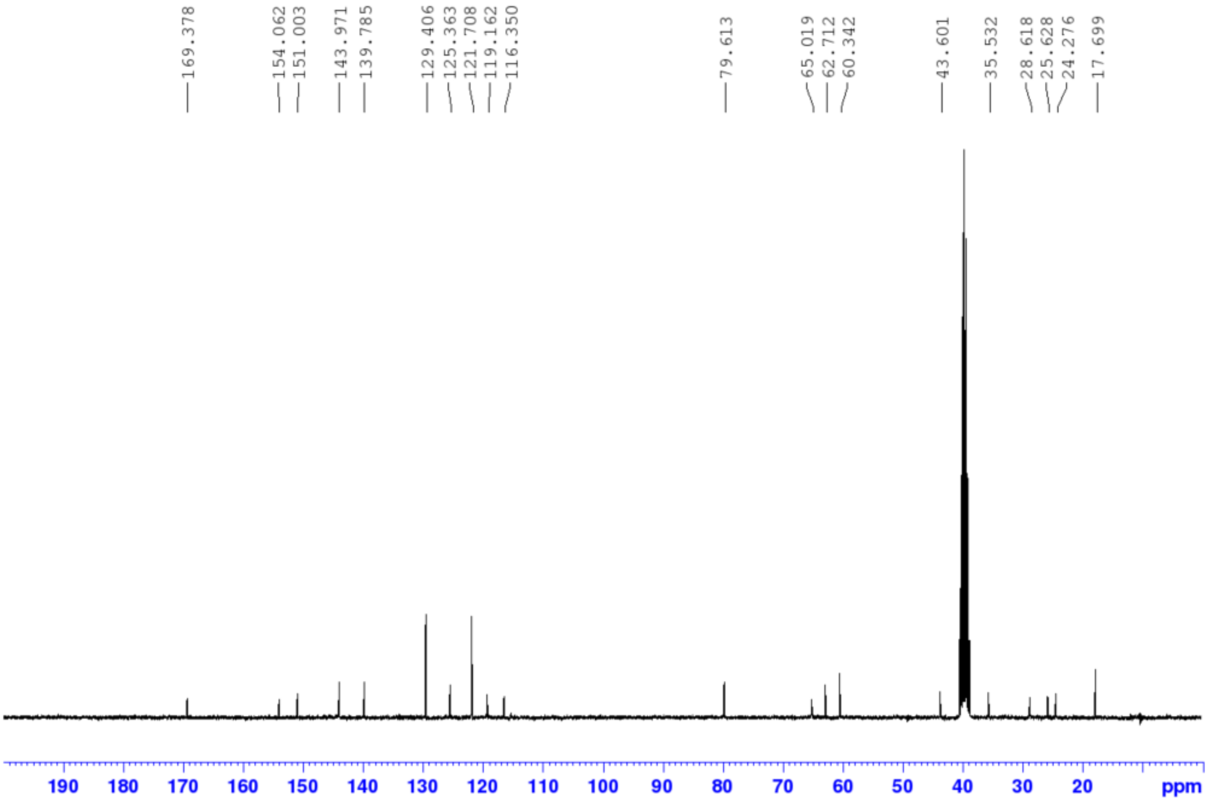

isobutyl N-hydroxy-N-[(1S,2S,4R,7R,11S)-4-methyl-8,12-dimethylene-13-oxo-3,14-dioxatricyclo[9.3.0.02,4]tetradecan-7-yl]carbamate (**18**)

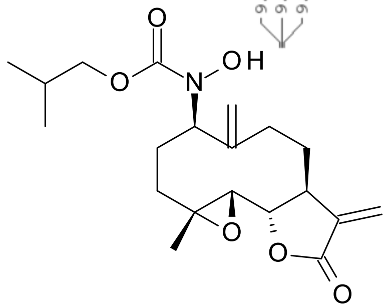

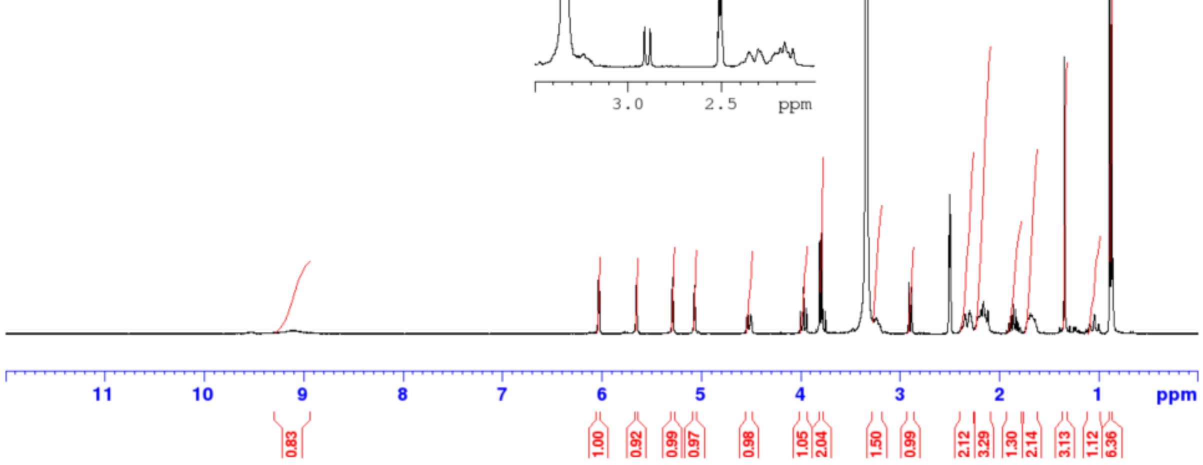

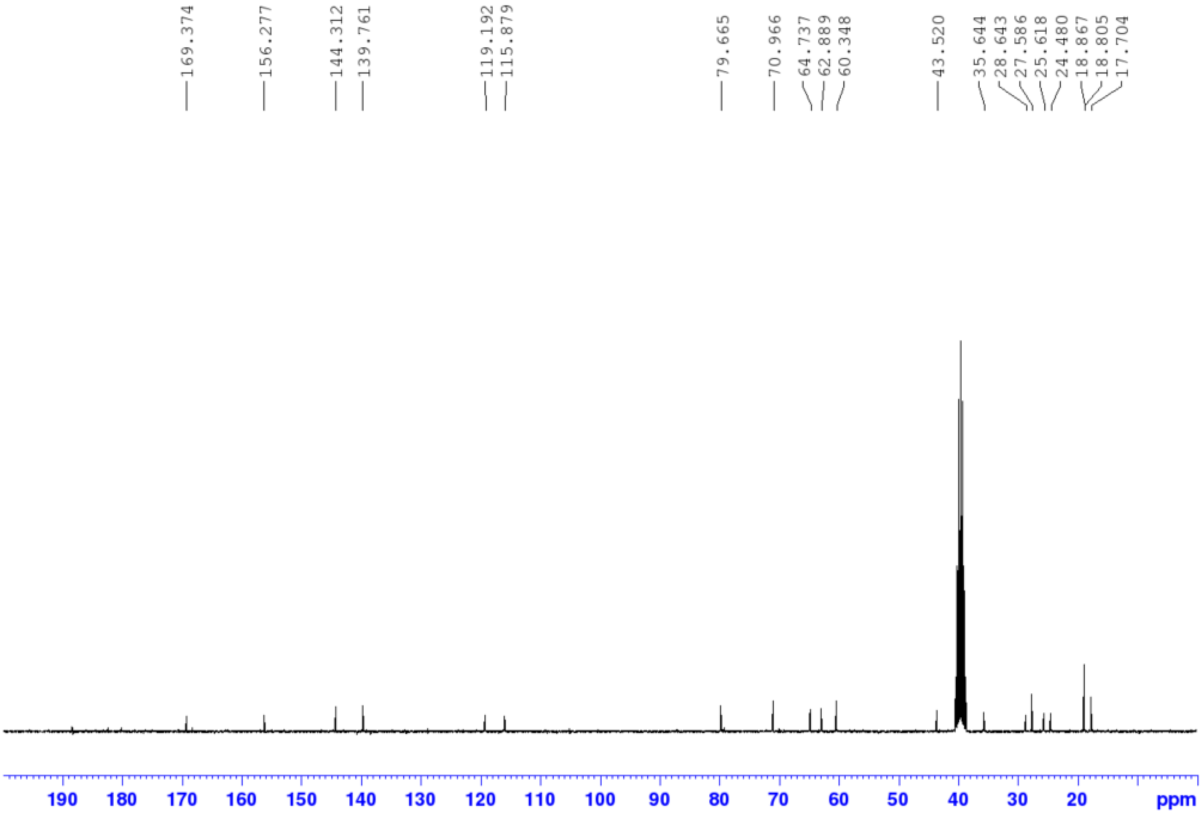

ethyl N-hydroxy-N-[(1S,2S,4R,7R,11S)-4-methyl-8,12-dimethylene-13-oxo-3,14-dioxatricyclo[9.3.0.02,4]tetradecan-7-yl]carbamate (**19**)

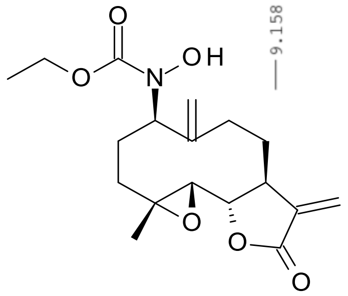

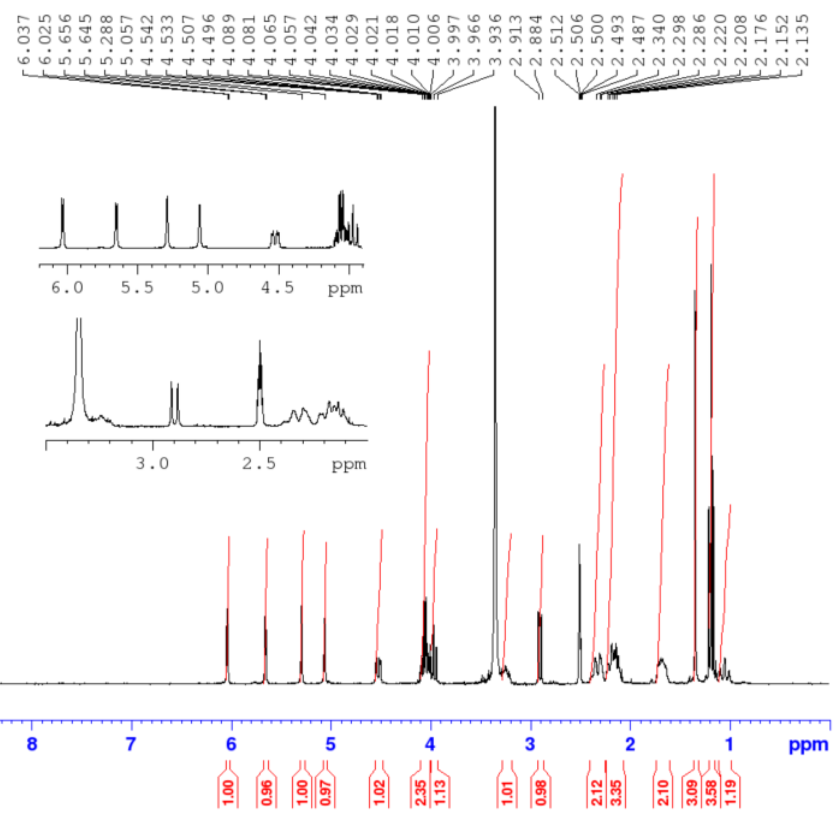

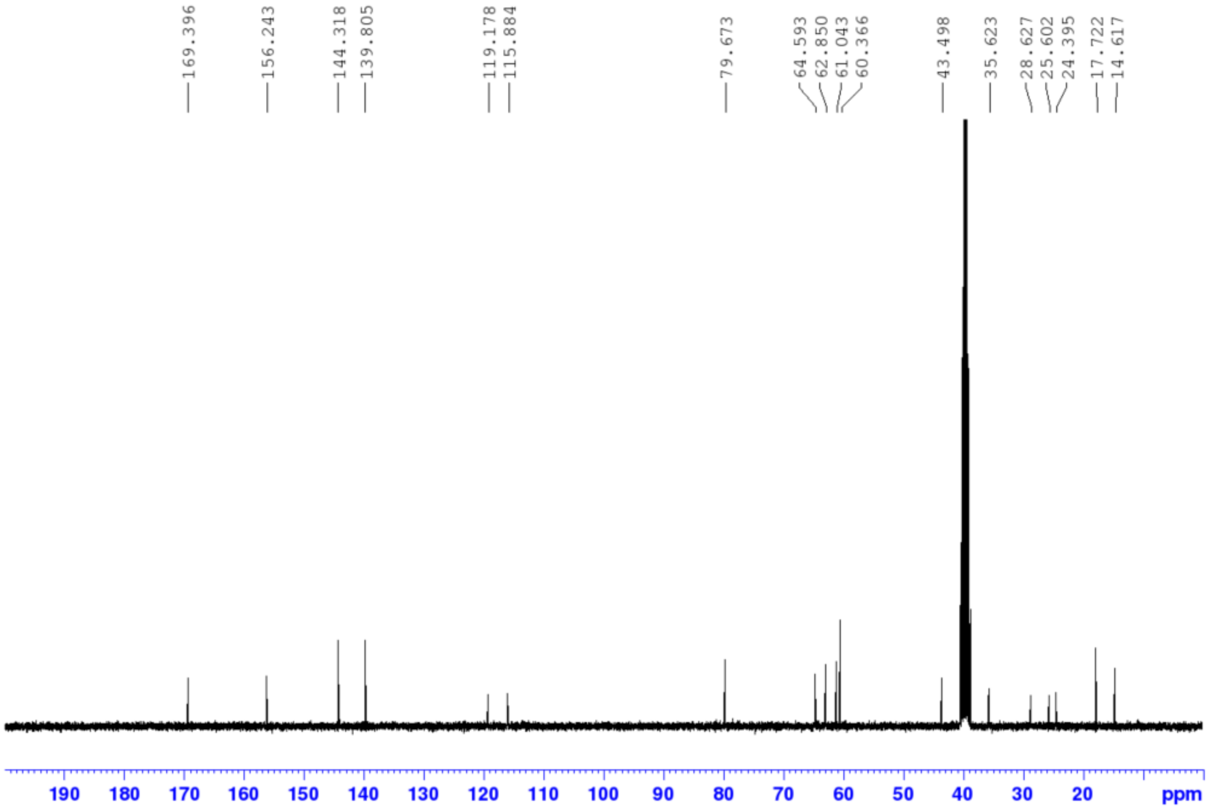

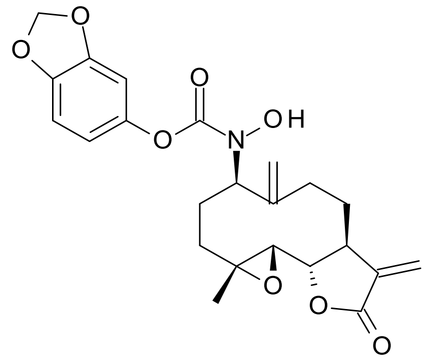

1,3-benzodioxol-5-yl N-hydroxy-N-[(1S,2S,4R,7R,11S)-4-methyl-8,12-dimethylene-13-oxo-3,14-dioxatricyclo[9.3.0.02,4]tetradecan-7-yl]carbamate (**20**)

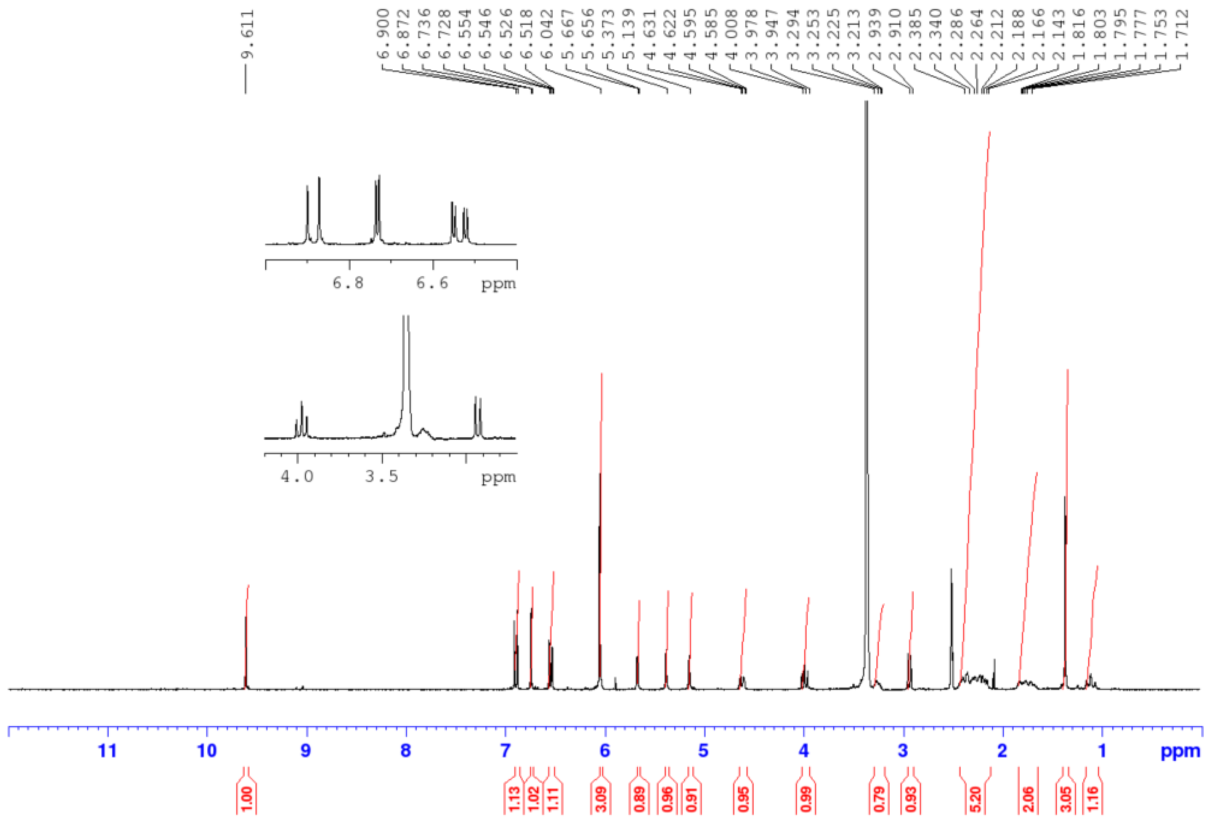

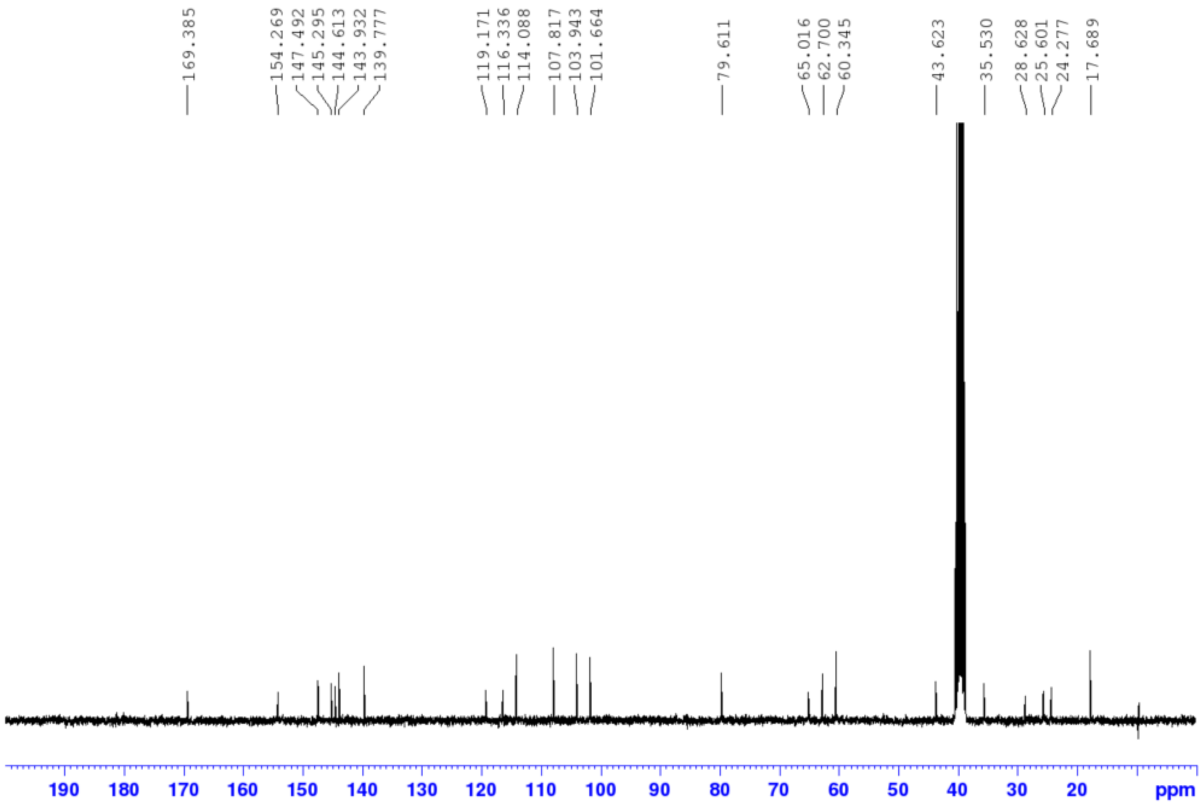
169.385
154.269
147.492
145.295
144.613
143.932
139.777
119.171
116.336
114.088
107.817
103.943
101.664
79.611
65.016
62.700
60.345
43.623
35.530
28.628
25.601
24.277
17.689
190
180
170
160
150
140
130
120
110
100
90
80
70
60
50
40
30
20
ppm

2-methoxyethyl N-hydroxy-N-[(1S,2S,4R,7R,11S)-4-methyl-8,12-dimethylene-13-oxo-3,14-dioxatricyclo[9.3.0.02,4]tetradecan-7-yl]carbamate (**21**)

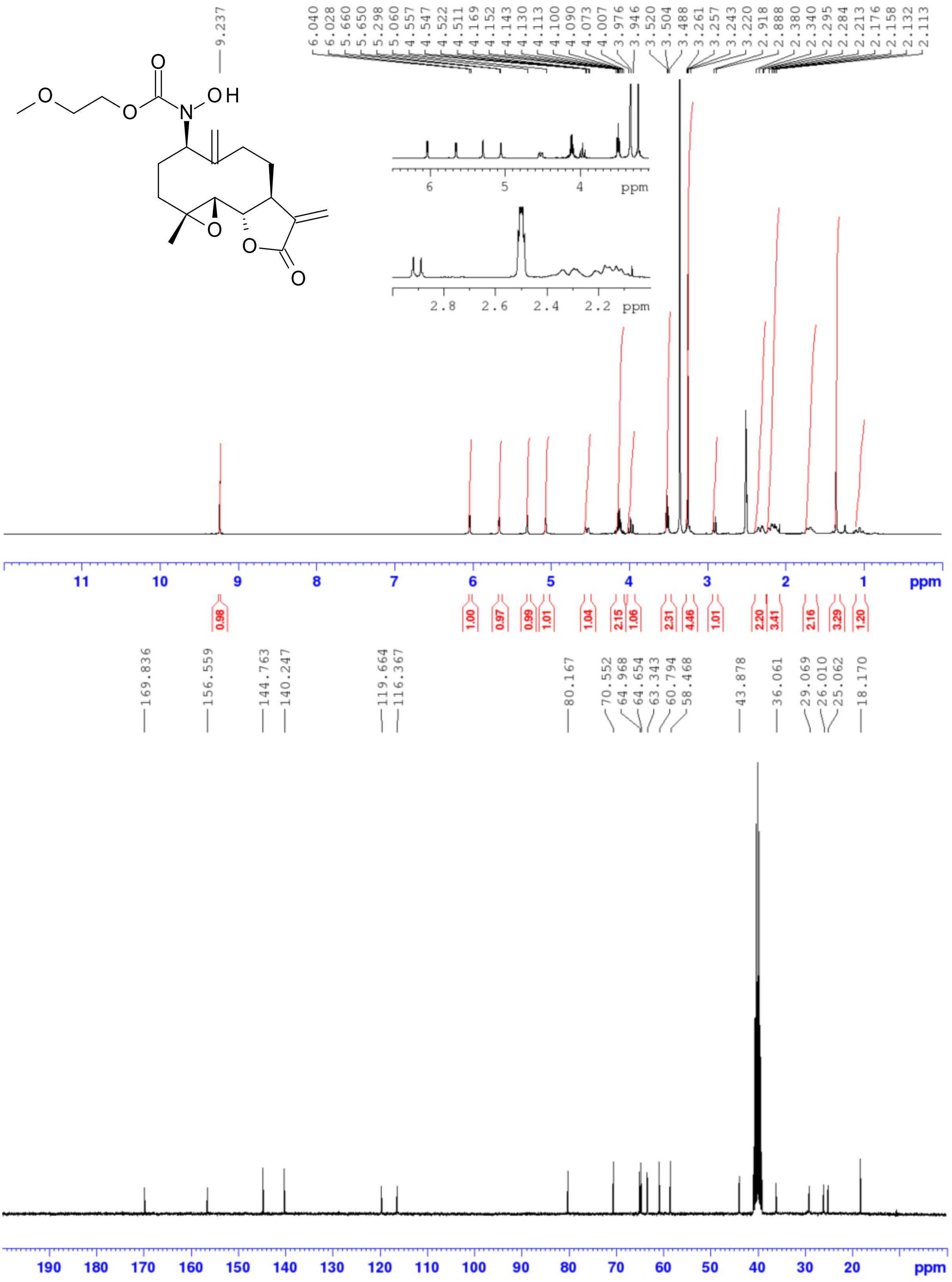

tetrahydropyran-4-yl N-hydroxy-N-[(1S,2S,4R,7R,11S)-4-methyl-8,12-dimethylene-13-oxo-3,14-dioxatricyclo[9.3.0.02,4]tetradecan-7-yl]carbamate (**22**)

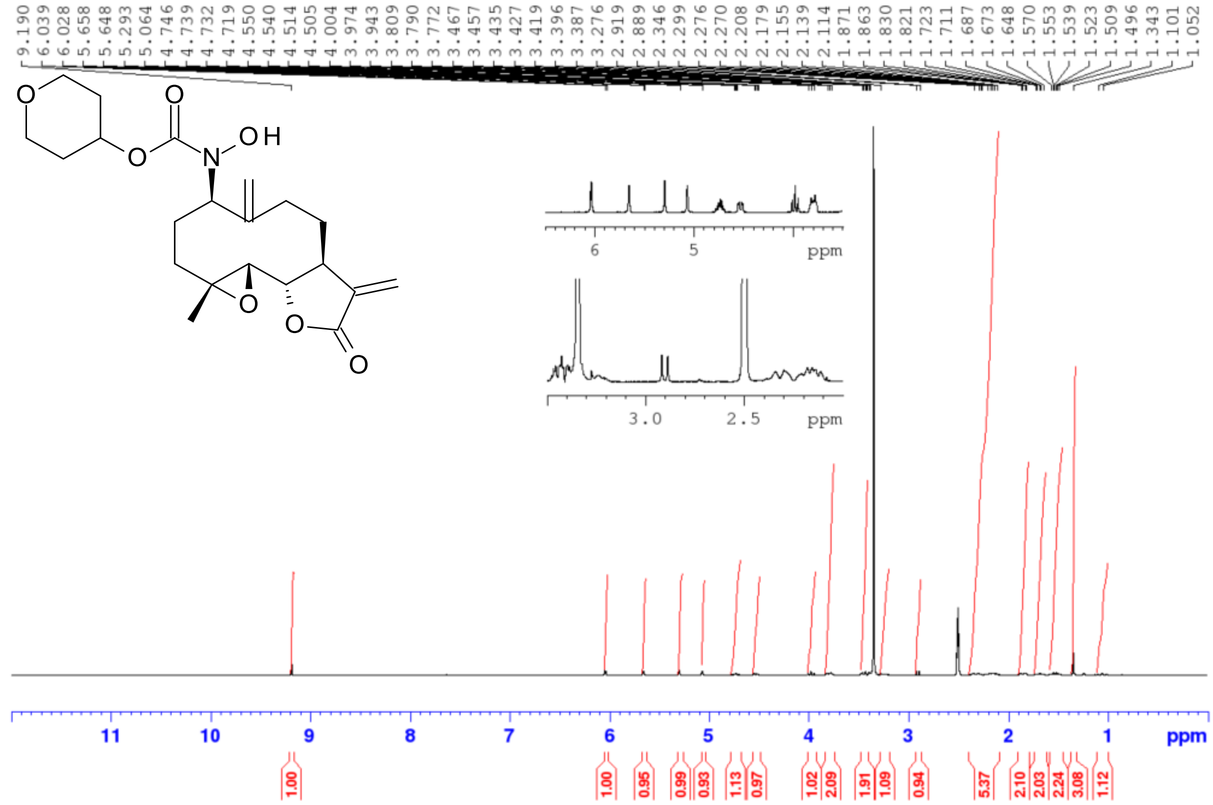

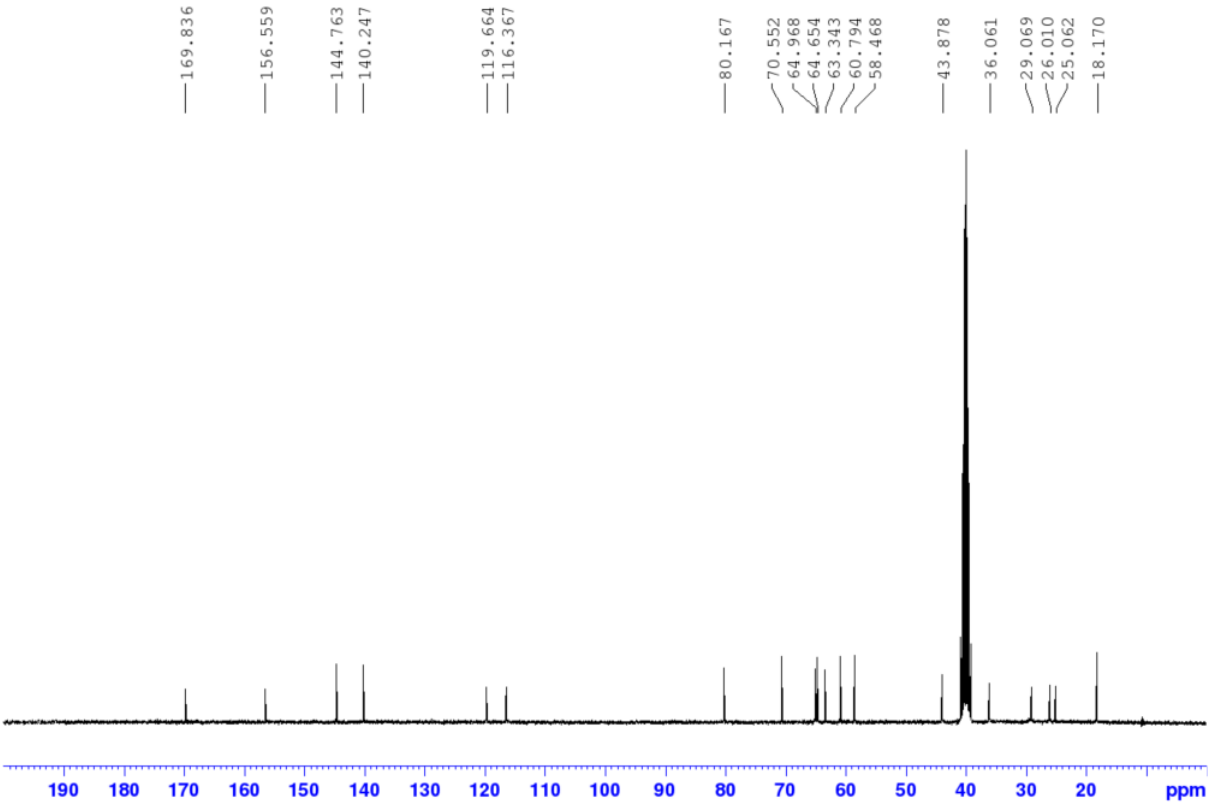

(1S,2S,4R,7Z,11S)-7-hydroxyimino-4-methyl-8,12-dimethylene-3,14-dioxatricyclo[9.3.0.02,4] tetradecan-13-one (**23**)

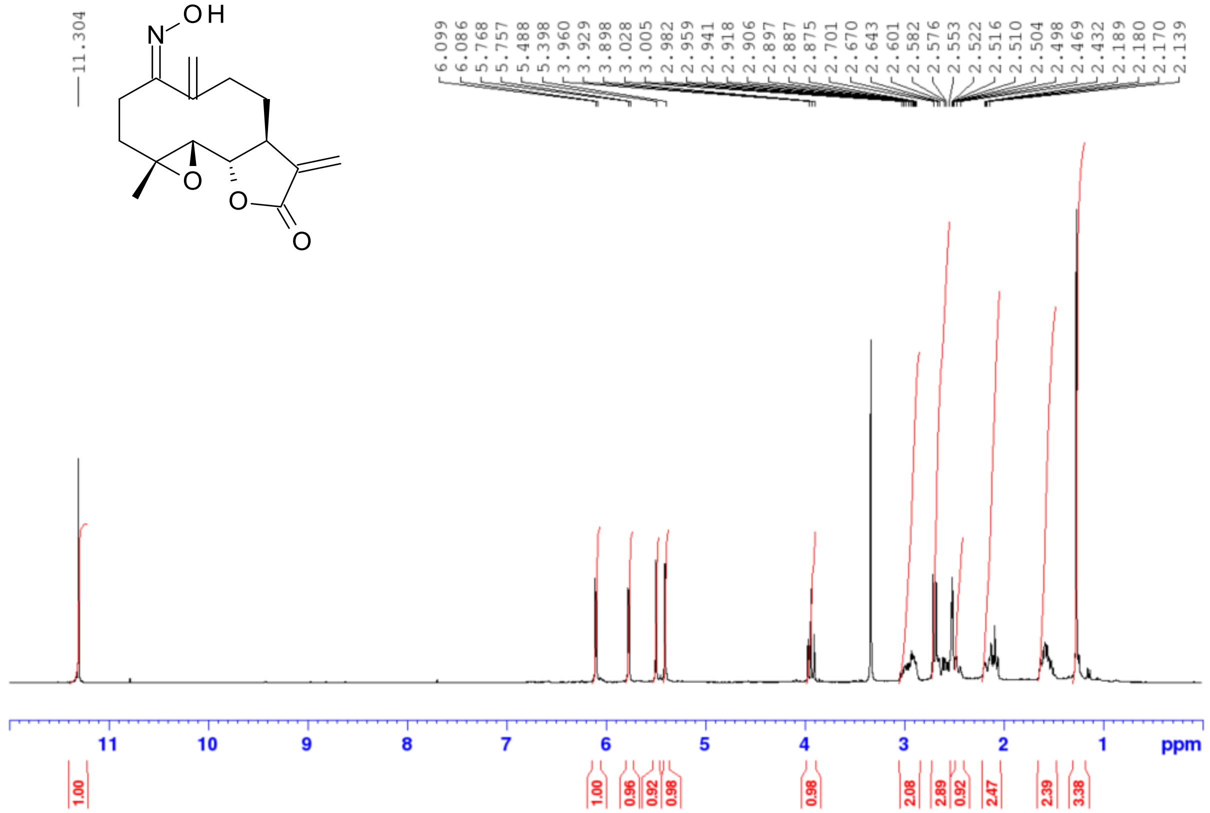

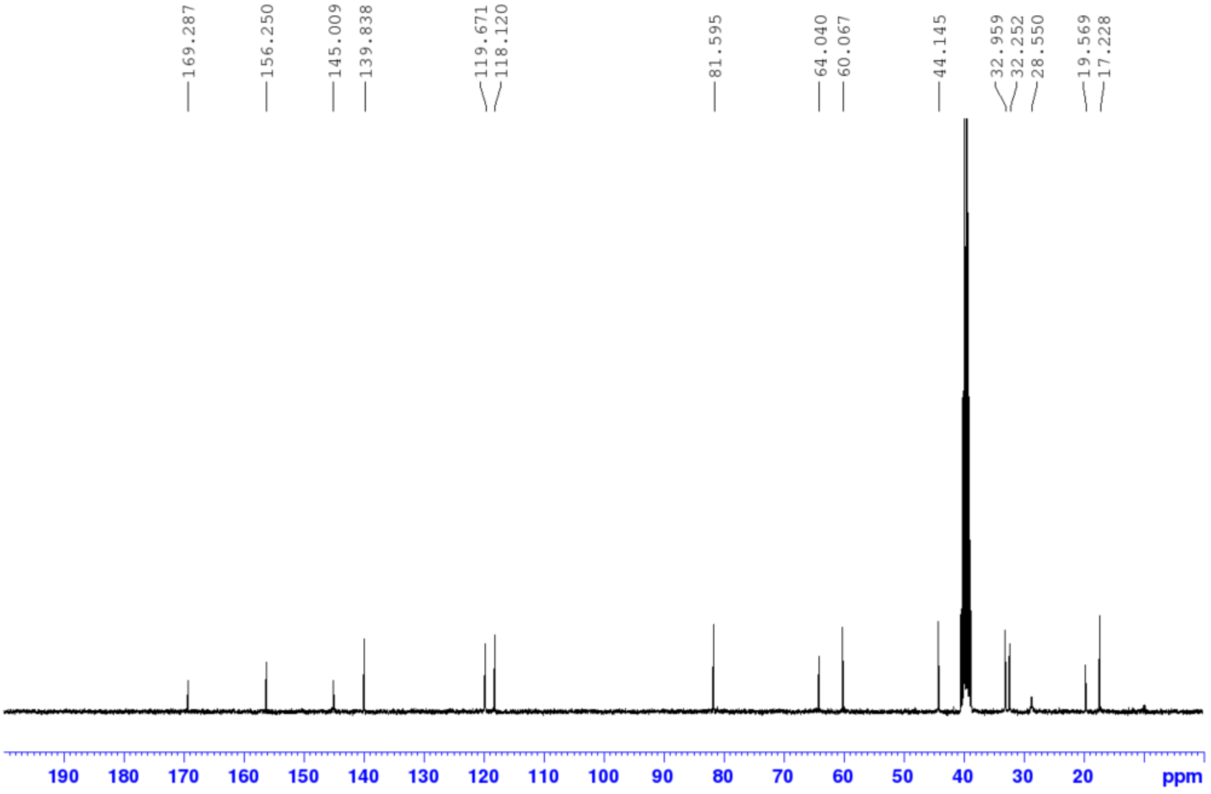

(1S,2S,4R,7E,11S)-4,8,12-trimethyl-3,14-dioxatricyclo[9.3.0.02,4]tetradec-7-en-13-one (**26)**

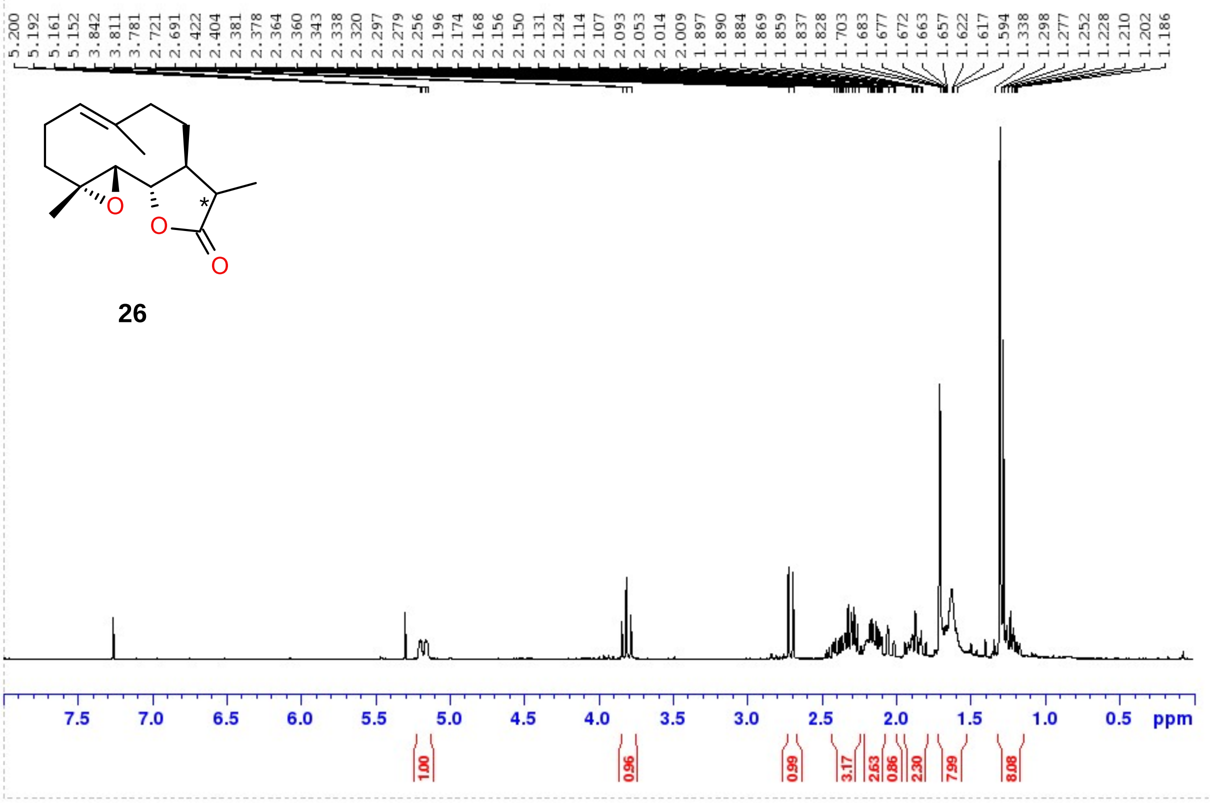

[1] N-Sulfonyloxy Carbamates as Reoxidants for the Tethered Aminohydroxylation Reaction n.d. https://doi.org/10.1021/ja057389g.
[2] Iron-Catalyzed Arene C–H Amidation Using Functionalized Hydroxyl Amines at Room Temperature n.d. https://doi.org/10.1021/acscatal.8b02939.
[3] Beier P, Mindl J, Štěrba V, Hanusek J. Kinetics and mechanism of base-catalysed degradations of substituted aryl-N-hydroxycarbamates, their N-methyl and N-phenyl analogues. Org Biomol Chem 2004;2:562–9. https://doi.org/10.1039/B310454K.
[4] Alkyl 4-Chlorobenzoyloxycarbamates as Highly Effective Nitrogen Source Reagents for the Base-Free, Intermolecular Aminohydroxylation Reaction n.d. https://doi.org/10.1021/jo1018816.
